# Biomolecular changes that occur in the antennal gland of the giant freshwater prawn (*Machrobrachium rosenbergii*)

**DOI:** 10.1371/journal.pone.0177064

**Published:** 2017-06-29

**Authors:** Utpal Bose, Thanapong Kruangkum, Tianfang Wang, Min Zhao, Tomer Ventura, Shahida Akter Mitu, Mark P. Hodson, Paul N. Shaw, Prasert Sobhon, Scott F. Cummins

**Affiliations:** 1 Genetic, Ecology and Physiology Centre, Faculty of Science, Health, Education and Engineering, University of the Sunshine Coast, Maroochydore DC, Queensland, Australia; 2 Metabolomics Australia, Australian Institute for Bioengineering and Nanotechnology, The University of Queensland, Brisbane, Queensland, Australia; 3 Department of Anatomy, Faculty of Science, Mahidol University, Bangkok, Thailand; 4 Center of Excellence for Shrimp Molecular Biology and Biotechnology (Centex Shrimp), Faculty of Science, Mahidol University, Bangkok, Thailand; 5 S chool of Pharmacy, The University of Queensland, Queensland, Australia; 6 Faculty of Allied Health Sciences, Burapha University, Chonburi, Thailand; Shanghai Ocean University, CHINA

## Abstract

In decapod crustaceans, the antennal gland (AnG) is a major primary source of externally secreted biomolecules, and some may act as pheromones that play a major role in aquatic animal communication. In aquatic crustaceans, sex pheromones regulate reproductive behaviours, yet they remain largely unidentified besides the *N*-acetylglucosamine-1,5-lactone (NAGL) that stimulates male to female attraction. In this study, we used an AnG transcriptome of the female giant freshwater prawn (*Macrobrachium rosenbergii*) to predict the secretion of 226 proteins, including the most abundantly expressed transcripts encoding the Spaetzle protein, a serine protease inhibitor, and an arthropodial cuticle protein AMP 8.1. A quantitative proteome analysis of the female AnG at intermolt, premolt and postmolt, identified numerous proteins of different abundances, such as the hemocyanin subunit 1 that is most abundant at intermolt. We also show that hemocyanin subunit 1 is present within water surrounding females. Of those metabolites identified, we demonstrate that the NAGL and *N*-acetylglucosamine (NAG) can bind with high affinity to hemocyanin subunit 1. In summary, this study has revealed components of the female giant freshwater prawn AnG that are released and contribute to further research towards understanding crustacean conspecific signalling.

## Introduction

Crustaceans attract a mate through visual, acoustic, vibratory or waterborne chemical (pheromone) mechanisms. The pheromone cues are thought to play a major role in the mating and courtship behaviours as females enter their reproductive premolt stage [1,2]. In many crustaceans, such chemical cues may derive from the antennal (or green) gland (AnG), a urine-producing organ that is located at the base of the antenna, consisting of a pair of nephropores with an opening located antero-ventrally [3]. Consistent with a role in conspecific signalling, urine released by female lobsters (*Homarus americanus* and *Carcinus maenas*) at different molting stages can promote male courtship responses, suggesting the AnG to be a source of sex pheromones [4,5]. Additionally, urine from premolt females of the three spot swimming crab (*Portunus sanguinolentus*), can induce male sexual behaviour, and this behaviour is lost when the release of female urine is blocked [6].

Many species of true crabs (infraorder Brachyura) have a terminal reproductive molt, making them an excellent model organism for pheromone research since the terminal reproductive molt is distinct from previous growth-related molts [7]. This includes the giant freshwater prawn *Macrobrachium rosenbergii*, where sexually mature females can undergo continuous growth molt or reproductive molt phases. The intricate social hierarchy in *M*. *rosenbergii* has been thoroughly documented in a series of studies [reviewed by (Ventura and Sagi, 2012 [8])], showing that females molt before mating and seek the protection of a dominant male in their vicinity during this vulnerable postmolt stage. Within a few days, the female will be fertilised, either by this male, by other dominant/subordinate males that are rivals, or by small sneak-copulating males, or by a combination [9,10]. These behaviours, together with it being the most highly cultured freshwater prawn worldwide [8], makes it an ideal organism to study pheromone release.

Attempts to uncover the identity of the crustacean female urine pheromone cue(s), have revealed numerous small biomolecules, including pentadecane, heptadecane and dichloroacetic acid, which are all abundant in the urine of the premolt and postmolt female blue swimming crab, *Portunus pelagicus* [11]. The most compelling evidence of a sex pheromone has been demonstrated from research on the blue crab, *Callinectes sapidus*, where *N*-acetylglucosamino-1,5-lactone (NAGL) present in the female AnG urine at intermolt and premolt, can activate physiological and behavioural responses in conspecifics [12]. Supporting a key role for the AnG in sex pheromone release, a recent transcriptomic screen of several tissues from the Eastern spiny lobster (*Sagmariasus verreauxi*), found that the most significantly biased transcript expression between males and females is within the AnG (other than the gonad) [13]. However, a large-scale analysis of this gland utilising a combined approach of transcriptomic, proteomic and metabolomic methodologies has not been performed to date. Such a resource would be helpful to assess the potential existence, synthesis and source of pheromones.

In this study, we investigated the female *M*. *rosenbergii* AnG transcriptome followed by *in silico* protein secretome prediction. We then performed mass spectral quantitative identification of proteins within the AnG at intermolt, premolt and postmolt. High resolution Ultra High Pressure Liquid Chromatography-Quadrupole Time of Flight-Mass Spectrometry (UHPLC-QToF-MS) combined with multivariate/chemometric approaches were applied to the same stages to reveal the metabolites present, highlighting some metabolites that are clearly molt stage specific. We propose that *N*-acetylglucosamine (NAG) and NAGL binds to *M*. *rosenbergii* haemocyanin subunit 1, which may contribute to conspecific pheromone communication.

## Materials and methods

### Ethics statement

All animal handling protocols are approved by the Experimental Animal Ethics Committee, Faculty of Science, Mahidol University, Thailand.

### Animal and antennal gland collection

Adult female *M*. *rosenbergii* (6–7 months) (average body weight 35–45 g) were obtained from Pran-Nok market (Bangkok, Thailand) and maintained in ventilated aquaria tanks for one week before tissue collection. Pathogen contamination and health were evaluated visually before selection. All prawns were fed once a day with commercial pellets (OMEG 1704S, Betagro, Thailand). Three stages (premolt, postmolt, intermolt; based on a previous report [10]), were evaluated. Animals were anesthetised on ice, quickly culled, then pairs of AnGs (n = 30) were dissected out. Then samples were either (i) stored at -80°C for RNA isolation, or (ii) freeze-dried (Thermo Supermodulyo-230, Thermo Scientific, USA) for protein and metabolite analysis.

### Illumina RNA-seq and annotation of the antennal gland

Approximately 18 AnGs from all three molting stages of female prawns (6 glands each) were washed with Millipore water, homogenised in TRIzol reagent (Invitrogen, Victoria, Australia), and processed following the manufacturer’s protocols. Purified total RNA was then dissolved in 50 μL of warmed RNase-free water and pooled. The quantity and quality of pooled total RNA was assessed using UV spectrophotometry (NanoDrop ND-1000, NanoDrop Technologies, DE, USA). Approximately 2 μg of total RNA was then used for RNA-seq using the Illumina sequencing platform following the generation of a cDNA library (BGI, Shenzhen, China). Before transcriptome assembly and mapping, filters were implemented to remove low quality reads and adaptor sequences.

To predict gene function and identify putative conserved protein domains to the unigene set, we compared translated protein sequences deduced from our contigs to multiple functional domain databases using RPS-BLAST and Blast2GO. Initially, the entire transcribed sets were compared to the SMART, COG conserved domains database, Protein Family Database (Pfam), and CDD databases using RPS-BLAST with no expected value threshold cut-off; only matches with an expected value less than 1×10^−10^ were considered in further analyses. We then mapped our contigs for gene ontology (GO) searching of the GO database and Blast2GO platform [14]. A conservative set of contigs was obtained using the bioinformatics suite above, and contigs were run through a 6-frame ORF filtering criteria (ExPASy-Translate tool [http://web.expasy.org/translate/] and NCBI ORF finder [http://www.ncbi.nlm.nih.gov/gorf/gorf.html]) to provide a *M*. *rosenbergii* AnG protein database.

### Sample preparation for mass spectrometry

To isolate proteins for proteomics analysis, freeze-dried AnG samples (7 glands each) were ground to a powder under liquid nitrogen in a mortar, then quickly weighed and homogenised in extraction buffer (90% methanol, 9% glacial acetic acid in deionized water) in a 1:5 w:v ratio. Crude extracts were then sonicated with three pulses, 30 s each, and centrifuged for 20 min (16,000 x g, 4°C). The supernatant was collected and lyophilised.

For the preparation of AnG sample for metabolomics analysis, freeze-dried AnG tissue samples (7 glands each) were placed into 1.5 mL of prechilled MeOH: water (1:1), then homogenised using a Qiagen TissueLyser (25 Hz, 5 min cycle). Samples were then centrifuged at 16,000 x g for 10 min. Finally, the supernatant was collected, freeze-dried and stored at -80°C. Freeze-dried samples were re-suspended in 15% of the original volume by adding 30 μL methanol and then 120 μL of MilliQ water (Millipore, Bedford, MA, USA) to produce a 20:80 methanol:water solution. The extract solution was stored at -80°C until use.

For the preparation of prawn-conditioned water, female *M*. *rosenbergii* at intermolt (n = 6) and molting (includes premolt and postmolt; n = 6) stages were separately cultured in individual tanks containing 10 L of reverse osmosis purified water. After 3 h incubation, the prawn-conditioned water was filtered with 90 mm Whatman filter paper to remove debris. Prawn-conditioned water was then freeze-dried (Thermo Supermodulyo-230, Thermo Scientific, USA).

### Nano HPLC-ESI-Triple TOF peptide identification in antennal gland

Purified AnG extracts were desalted using Ziptip C18 (Millipore, Australia) then analyzed by LC-MS/MS on a Shimadzu Prominence Nano HPLC (Kyoto, Japan) coupled to a Triple TOF 5600 mass spectrometer (ABSCIEX, Concord, Canada) equipped with a nano-electrospray ion source, as previously described [15]. Briefly, approximately 6 μL of each extract was injected and de-salted on the trap column before entering the HPLC column (Agilent Technologies, Australia) for mass spectrometry analysis. The mass spectrometer acquired 500 ms full scan TOF-MS data followed by 20 by 50 ms full scan product ion data. Full scan TOFMS data was acquired over the mass range 350–1800 and for product ion MS/MS 100–1800. Ions observed in the TOF-MS scan exceeding a threshold of 100 counts and a charge state of +2 to +5 were set to trigger the acquisition of product ion spectra. The data were acquired and processed using Analyst TF 1.5.1 software (ABSCIEX, Canada).

Fragmentation data were analysed by PEAKS v7.0 (BSI, Canada) software. Sequences of peptides were determined manually and by comparing the fragmentation patterns with those predicted from the *M*. *rosenbergii* AnG transcriptome protein database. Search parameters were as follows: no enzyme was used, variable modifications included methionine oxidation, conversion of glutamine/glutamate to pyroglutamic acid, deamidation of asparagine and peptide amidation. Precursor mass error tolerance was set to 20 ppm and a fragment ion mass error tolerance was set to 0.1 Da. The false discovery rate (FDR) was set to ≤ 1%, and the individual peptide ion score [-10*Log(p)] was calculated accordingly, where p is the probability that an observed match is a random event. Proteins and their supporting peptides were obtained and analysed.

### Nano HPLC-ESI-Triple TOF peptide identification in prawn-conditioned water

Lyophilised prawn-conditioned water was resuspended in 0.1% v/v aqueous trifluoroacetic acid (TFA), then centrifuged (10 min at 12,000 rpm). The supernatant was transferred into a fresh tube before desalting using Sep-Pak C18 cartridges (Waters Associates, Milford Mass, USA). Biomolecules were eluted using 60% v/v acetonitrile (Sigma-Aldrich, Sydney, Australia) in 0.1% v/v aqueous TFA (Sigma-Aldrich, USA) and then lyophilised. Biomolecules were resuspended in 0.1% v/v aqueous TFA in MilliQ water. Aliquots (100 μL) of each were injected onto 300SB-C18 Zorbax column stable bond analytical 4.6 mm x 250 mm at 1 mL/min. Column equilibration was performed by a linear gradient of 95–0% solvent B [0.1% TFA in acetonitrile] continuously elution over a period of 30 min (1 mL/min), followed by a steeper gradient from 0% to 60% solvent B. The eluate was collected every 1 min continuously to 60 min. After that solvent B was increased by gradient to 95% over 15 min to wash the column and then returned to 0% solvent B for equilibration prior to the next sample injection. The protein fractions of three profiles were selected for lyophilisation and analysed with mass spectrometry LC-ESI-Triple TOF, as described above.

### Metabolite profiling with high-resolution accurate mass spectrometry (HRAMS)—UHPLC-QToF-MS analysis

Samples were analysed using an Agilent UHPLC-QToF-MS system (Agilent Technologies, Santa Clara, CA, USA) comprising a 1290 UHPLC coupled to a 6520 Accurate-Mass Quadrupole Time-of-Flight Mass Spectrometer (QToF-MS, Agilent Technologies, Santa Clara, CA, USA) in positive and negative mode from m/z 100 to 1700 for all samples at a scan rate of 0.8 cycles/s. Instrument resolution was 9000–11,700 across the data acquisition range. This mass range enabled the inclusion of two reference compounds: a lock mass solution including purine (C_5_H_4_N_4_) at *m/z* 121.050873, 10 μmol.L^-1^) and hexakis (1H, 1H, 3H-tetrafluropentoxy)-phosphazene (C_18_H_18_O_6_N_3_P_3_F_24_ at *m/z* 922.009798, 2 μmol.L^-1^). Chromatographic separation was achieved using a Phenomenex Gemini-NX C18 HPLC column (150 mm × 2.0 mm, 3 μm, Phenomenex, Lane Cove, NSW, Australia). The mobile phase consisted of (A) water containing 5 mM ammonium acetate (UniVar Analytical reagents, Sydney, Australia) and (B) acetonitrile (LabScan Analytical Science, Taren Point, Australia). In all HPLC runs the elution gradient started at 80% A: 20% B increasing to 0% A: 100% B over a period of 40 min, followed by a 5 min hold and 20 min re-equilibration period. A sample volume of 20 μL was injected for each HPLC run. The HPLC run contained blanks and pooled QC samples [16] intercalated throughout the HPLC run to control for any acquisition-dependent variation. The samples and standards were filtered using a 0.2 μm PTFE membrane filter (Phenomenex, Torrance, CA, USA) before analysis.

Data analysis was performed using Agilent MassHunter Qualitative software (Version B.05.00). The Molecular Feature Extractor algorithm within MassHunter Qualitative analysis software was used to extract chemically qualified molecular features from the UHPLC-QToF-MS data files. For empirical formula generation, the Molecular Formula Generator algorithm was used. This algorithm uses a wide range of MS information, for instance accurate mass measurements, adduct formation, multimer formation and isotope patterns to generate a list of candidate compounds. The maximum elemental composition C_60_H_120_O_30_N_30_S_5_Cl_3_Br_3_ was used to generate formulae. Molecular formula generation (MFG) can automatically eliminate unlikely candidate compounds and rank the putative molecular formulae according to their mass deviation, isotopic pattern accuracy and elemental composition. Samples from different molting stages were evaluated separately by multivariate analysis. Feature-extracted sample files were transferred into Agilent GeneSpring software version 12.0 (Agilent Technologies, Santa Clara, CA, USA) for alignment and to compile data matrix.

### Chemometric analyses and identification of compounds

AnG metabolite samples from female postmolt, intermolt and premolt were evaluated separately by multivariate statistical analysis. Feature-extracted sample files were transferred into Agilent GeneSpring software version 12.0 (Agilent Technologies, Santa Clara, CA, USA) for alignment and to compile the data matrix. The data matrix (positive mode: 577 variables; 8 observations—one observation from post molting time omitted from the analysis due to unsuccessful extraction/acquisition; negative mode: 297 variables) was imported into SIMCA-P+ version 13.0 (MKS Umetrics AB, Umeå, Sweden) for multivariate data analysis. Primarily principal component analysis (PCA) and orthogonal projection to latent structures-discriminant analysis (OPLS-DA) was used to interrogate the dataset. For more information relating to these methods see [16]. Data were pre-treated by log10 transformation and mean centring before analysis. Data were also analysed using pareto and unit variance scaling, but such scaling was deemed superfluous for feature selection in this dataset and thus only mean centred analysis/results are shown. For OPLS-DA models a minimum threshold of VIP>1 was used for variable selection (= “importance”) [17].

### Enzyme pathway annotation

Sequences of pathway enzyme genes were obtained from the National Center for Biotechnology Information (NCBI; www.ncbi.nlm.nih.gov), and then used as queries for tBLASTn searches of a *M*. *rosenbergii* assembled AnG transcriptome (SRA file SUB923189). BLASTp searches were performed using the CLC Main Workbench Version 6.0 with an e-value cut-off 10^−3^. Multiple sequence alignments were created with the Molecular Evolutionary Genetics Analysis (MEGA) software version 6.0 [18] and phylogenetic trees constructed using the neighbor-joining method with a minimum 1000 bootstrap replicates for node support. Sequence presentation and shading of multiple sequence alignments was performed using the LaTEX TEXshade package [19]. The SMART (Simple Modular Architecture Research Tool) was used to identify and annotate conserved domains present in individual proteins [20].

### Protein modelling

The initial conformation of the *M*. *rosenbergii* haemocyanin subunit 1 was built using SWISS-MODEL by sequence alignment with proteins with known 3D structures (template proteins). The structure with the highest quality estimation (QMEAN score) was chosen, and subjected to molecular dynamics simulation (MDS) using AMBER version 16 [21]. The structure was imported using the LEAP module of AMBER; the sequence segment(s) that miss-represented (usually at N- or C- terminus) due to the different sequence length of the template proteins, was built as a linear structure using LEAP and linked back to the corresponding positions. MDS was fully unrestrained and carried out in the canonical ensemble using the SANDER module. The ff14SB force field [22] was employed. Energy minimisation with 2500 steps was first performed to remove unfavourable contacts. The AMBER structure was then heated to 325K over 50 ps to avoid being kinetically trapped in local minima, then subjected to unrestrained MD simulations at 325K for the purpose of peptide equilibration. The structural information was sampled every 1 ps (i.e., 10,000 structures were calculated for 10 ns MD simulation). This MD simulation was continued until the root mean square deviation (RMSD) of structures within a reasonable long time range is stably at/less than 3~4Å, calculated using PTRAJ implemented in AMBER. Then the lowest energy structure can be determined, and considered as the representative of the conformations simulated over this period. Visualisation of the systems was effected using via VMD software [23]. The representative structure of haemocyanin was then used as the ‘receptor’, and its possible binding sites with the target ligands, NAG or NAGL, were calculated by PatchDock [24]. The structures of the ‘receptor’ protein (haemocyanin) and the ligand (either NAG or NAGL) in PDB format were imported to PatchDock, and the clustering RMSD was set to 3.0. The top ten best docking solutions, i.e., the complexes with highest scores, were exported for presentation.

## Results and discussion

### Summary of female *M*. *rosenbergii* AnG transcriptome

**Fig 1:**
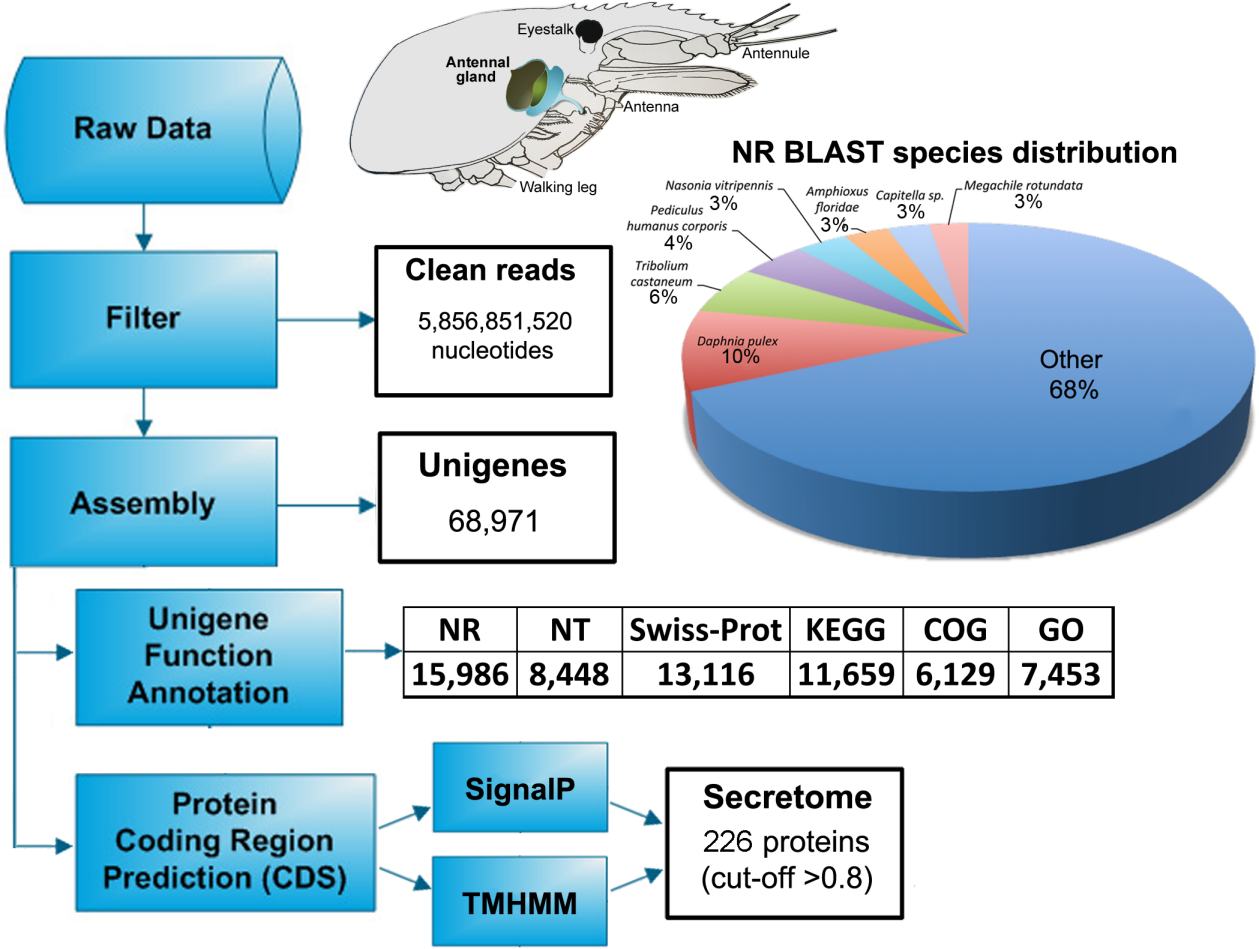
Workflow of *M*. *rosenbergii* antennal gland transcriptome preparation and analysis. The schematic of prawn head and cephalothorax shows the position of the antennal gland. NR, protein database; NT, nucleotide database; Swiss-prot, curated protein sequence database; KEGG, Kyoto Encyclopedia of Genes and Genomes; COG, Clusters of Orthologous Groups; GO, gene ontology.

The crustacean AnG is comparable to the kidney of higher vertebrates and Malphigian tubules in other arthropod sub-phyla. The AnG consists of one pair of excretory units, connected to an opening pore located bilaterally at the antennae base [25] (Fig 1), where they function to excrete nitrogenous wastes and for water-salt balance [26,27]. Within the intercellular gland space, haemolymph fluid is filtered through podocytes [25] where any essential haemolymph biomolecules are reabsorbed at the gland labyrinth, and waste urine is stored in the bladder until excretion [28].

RNA obtained from female *M*. *rosenbergii* AnG was sequenced to provide a total of 5.8 Gb clean nucleotide reads (SRA file SRP102747) that were assembled into a transcriptome library. A summary of transcriptome analysis is presented in Fig 1. Assembly resulted in 68,971 unigenes with a mean length of 560 nucleotides (N50 = 709), of which 64,395 encode for proteins >30 amino acids in length. *M*. *rosenbergii* AnG sequences were annotated against databases (NR, NT, Swiss-Prot, KEGG, COG and GO) using BLASTX (E-value < 0.00001). From the 68,971 consensus sequences, 15,986 had BLAST matches within the NR database. The sequence names and annotation information of all sequences are provided in S1 File. Species with the highest number of matches within the non-redundant (NR) database were the water flea *Daphnia pulex* (9.7%), red flour beetle *Tribolium castaneum* (5.8%) and body louse *Pediculus humanus corporis* (4.4%). The annotation rate in our study is comparable to those that have been reported in previous *de novo* transcriptome sequencing studies for crustaceans [13,29]. Of specific interest were those proteins predicted to be secreted extracellular; i*n silico* analysis of the AnG transcriptome-derived protein database revealed that 579 proteins contain N-terminal signal peptides, and of those 226 contain no transmembrane domain (S2 File).

Within this subset of predicted secreted proteins, those most highly expressed were the protein Spaetzle, and an arthrodial cuticle protein AMP 8.1. Upon microbial attack, Spaetzle, a member of the cysteine-knot family of growth factor and cytokine-like proteins, acts as a ligand that binds to the transmembrane receptor Toll (or Toll/interleukin 2 receptors) [30]. Binding initiates the production of antimicrobial peptides that act to defend against Gram-positive bacteria and fungi [31]. This Spaetzle-Toll pathway has been described in various crustaceans including the Chinese mitten crab [32], Chinese white shrimp, and the horseshoe crab [33]. The relatively high abundance of *Spaetzle* transcripts in the AnG of *M*. *rosenbergii* also suggests that the prawn Spaetzle may be activating a pathway for antimicrobial peptide production. This high expression may be a requirement for microbial protection during their stages of molting period; this is a time in which the animal’s movement is slow and prone to attack by microbes (and predators). The Eastern spiny lobster contains 6 Spaetzle isoforms, all of which change throughout metamorphosis [34]. In this situation, Spaetzle is possibly used to activate antimicrobial peptides, although here it may also contribute to embryonic dorso-ventral axis determination, as has been observed in *Drosophila* embryogenesis [31]. Spaetzle within the AnG more likely initiates a protective mechanism, due to the glands exposure to the surrounding environment.

Other genes represented within the *in silico* secretome list include *UDP-glucuronosyltransferase* 2B4, which encodes for an enzyme that is of major importance in the conjugation and subsequent elimination of potentially toxic xenobiotics and endogenous compounds [35]. Also, a *crustacyanin-like lipocalin* is detected. Lipocalins include a family of proteins that carry small hydrophobic biomolecules such as steroids, retinoids, lipids and pheromones [36,37]. The crustacyanin lipocalin binds astaxanthin within the crustacean carapace, as determined in the lobster *Homarus gammarus* [38]. The presence of these two components within the *M*. *rosenbergii* AnG is congruent with its role in the removal of waste products, and possibly in pheromone transport.

### Proteomic analysis of antennal gland and prawn-conditioned water

**Fig 2:**
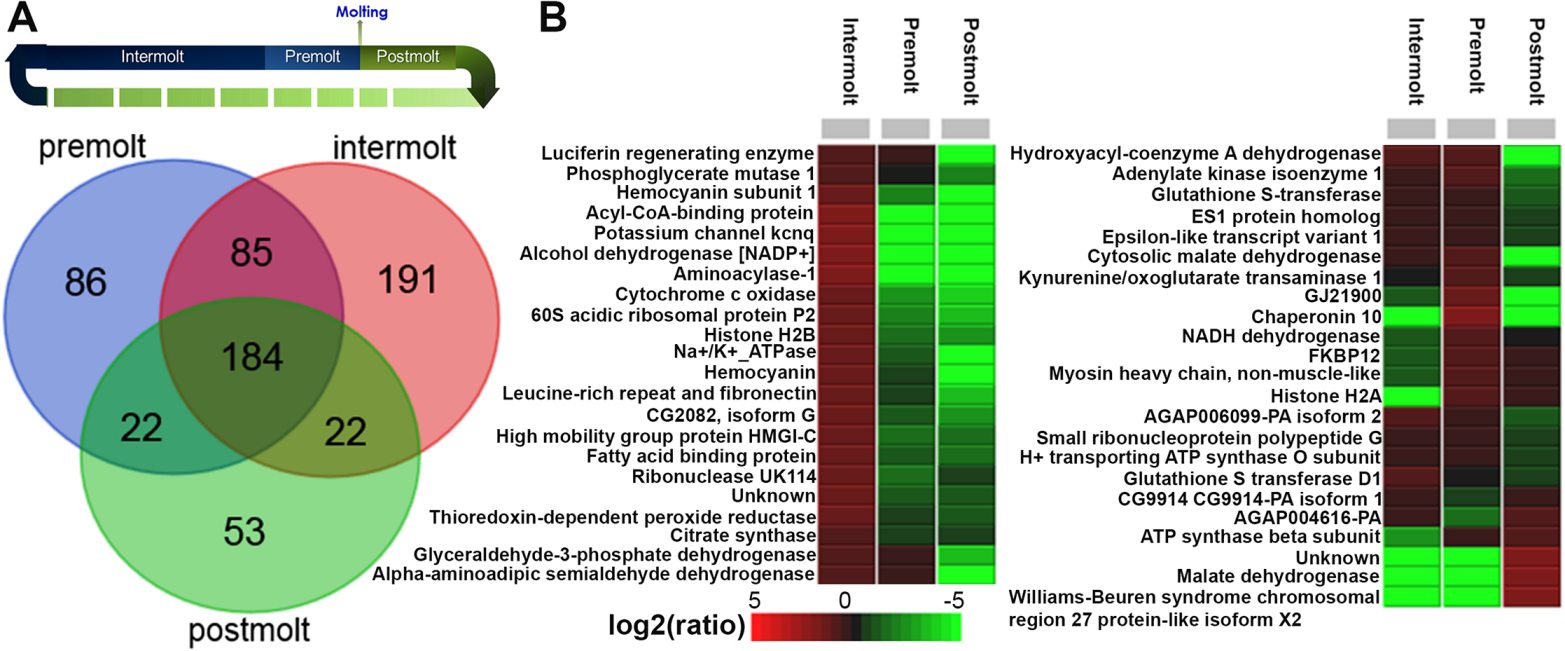
Analysis of proteins identified in the female *M*. *rosenbergii* antennal gland at premolt, intermolt and postmolt. (**A**) Molt timeline and Venn diagram showing distribution of proteins identified in different stages. (**B**) Heatmap showing relative abundance of proteins significantly different between stages.

Protein extraction of the female *M*. *rosenbergii* AnG followed by LC-MS/MS analysis was used to identify those proteins of differential abundance between intermolt, premolt and postmolt. In total, we identified 643 proteins, of which 184 were common to all stages (Fig 2A). Of the 226 predicted secreted proteins, 25 (11%) were identified in the proteomic analysis, including the Spaetzle and arthrodial cuticle protein AMP 8.1 (S2 File). Those proteins of significant differential abundance between each stage are shown in Fig 2B (see also, Table 1 and S3 File). Of those proteins secreted, haemocyanin subunit 1 was identified as relatively abundant within the AnG at intermolt.

**Table 1:** Summary of proteins differentially expressed in the *M*. *rosenbergii* antennal gland during intermolt, premolt and postmolt.

NR-annotation	Species match	NR-Evalue	Transcriptome database
Malate dehydrogenase	*Culex quinquefasciatus*	1.00E-128	Unigene45659_MrAnG
Glutathione S-transferase	*Eriocheir sinensis*	2.00E-76	Unigene11100_MrAnG
Fatty acid binding protein	*Scylla paramamosain*	1.00E-62	Unigene35155_MrAnG
Potassium channel kcnq	*Culex quinquefasciatus*	1E-13	CL4165_All
Aminoacylase-1	*Coturnix japonica*	1E-160	Unigene46324_All
Cytosolic malate dehydrogenase	*Daphnia pulex*	1.00E-137	Unigene37160_MrAnG
60S acidic ribosomal protein P2	*Scylla paramamosain*	3.00E-30	Unigene11482_MrAnG
Alpha-aminoadipic semialdehyde dehydrogenase	*Tribolium castaneum*	0	Unigene44798_MrAnG
High mobility group protein HMGI-C	*Exaiptasia pallida*	8.00E-04	Unigene12220_MrAnG
Williams-Beuren syndrome chromosomal region 27 protein-like isoform X2	*Lingula anatina*	8.00E-42	CL465.Contig1_MrAnG
Leucine-rich repeat and fibronectin	*Heterocephalus glaber*	2.00E-09	Unigene30749_MrAnG
AGAP004616-PA	*Anopheles gambiae*	1.00E-30	Unigene30712_MrAnG
GJ21900	*Drosophila virilis*	5.00E-52	Unigene11861_MrAnG
Alcohol dehydrogenase [NADP+]	*Zootermopsis nevadensis*	9.00E-114	Unigene42613_MrAnG
Cytochrome c oxidase subunit	*Aedes aegypti*	8.00E-27	Unigene40207_MrAnG
ATP synthase beta subunit	*Penaeus monodon*	0	CL473.Contig1_MrAnG
CG2082, isoform G	*Drosophila melanogaster*	2.00E-110	Unigene44527_MrAnG
Glyceraldehyde-3-phosphate dehydrogenase	*Cancer borealis*	0	Unigene42694_MrAnG
Acyl-CoA-binding protein	*Penaeus monodon*	1.00E-33	Unigene10645_MrAnG
Ribonuclease UK114	*Salmo salar*	1.00E-41	Unigene42192_MrAnG
CG9914 CG9914-PA isoform 1	*Tribolium castaneum*	8.00E-93	Unigene30770_MrAnG
Epsilon-like transcript variant 1	*Litopenaeus vannamei*	2.00E-136	Unigene39871_MrAnG
H+ transporting ATP synthase O subunit	*Antheraea yamamai*	1.00E-62	Unigene30446_MrAnG
FKBP12	*Bombyx mori*	5.00E-59	Unigene25136_MrAnG
Na+/K+_ATPase	*Exopalaemon carinicauda*	0	CL3624.Contig1_MrAnG
NADH dehydrogenase	*Centropages tenuiremis*	3.00E-34	Unigene47990_MrAnG
Hydroxyacyl-coenzyme A dehydrogenase	*Caligus rogercresseyi*	2.00E-149	Unigene24712_MrAnG
Thioredoxin-dependent peroxide reductase	*Ictalurus punctatus*	3.00E-111	Unigene35233_MrAnG
Chaperonin 10	*Scylla paramamosain*	7.00E-37	Unigene12955_MrAnG
Citrate synthase	*Aedes aegypti*	0	Unigene39332_MrAnG
Luciferin regenerating enzyme	*Lampyris turkestanicus*	3.00E-22	Unigene35730_MrAnG
AGAP006099-PA isoform 2	*Tribolium castaneum*	1.00E-94	Unigene43811_MrAnG
Phosphoglycerate mutase 1	*Harpegnathos saltator*	1.00E-118	Unigene39734_MrAnG
Adenylate kinase isoenzyme 1	*Trichinella nelsoni*	6.00E-83	Unigene34775_MrAnG
Unknown	N/A	N/A	Unigene48072_MrAnG
Histone H2A	*Athalia rosae*	3.00E-61	Unigene2032_MrAnG
Unknown	N/A	N/A	Unigene16324_All
ES1 protein homolog	*Strongylocentrotus purpuratus*	9.00E-75	CL5937.Contig1_MrAnG
Myosin heavy chain, non-muscle-like	*Megachile rotundata*	0	CL3591.Contig1_MrAnG
Haemocyanin subunit 1	*Macrobrachium nipponense*	0	CL2258.Contig1_All
Aminoacylase-1-like isoform 1	*Gallus gallus*	1.00E-140	Unigene16324_All
Histone H2B	*Rhynchosciara americana*	1.00E-60	Unigene1432_All
Haemocyanin	*Macrobrachium nipponense*	0	Unigene8041_All
Kynurenine/oxoglutarate transaminase 1	*Pediculus humanus corporis*	2.00E-157	CL2115.Contig1_All
Small ribonucleoprotein polypeptide G	*Cherax quadricarinatus*	1.00E-32	Unigene4833_All
Glutathione S-transferase D1	*Procambarus clarkii*	1.00E-64	Unigene770_All

LC-MS/MS was then used to analyse proteins present within water that had been conditioned with intermolt and molting female *M*. *rosenbergii*. Those proteins identified are summarised in Table 2, and include the haemocyanin subunit 1. Haemocyanins are large, multi-subunit molecules containing highly conserved copper-based oxygen binding sites. In many arthropod and molluscan species, oxygen is transported within the circulatory system by hemocyanins [39] and this sustains metabolic requirements. In addition to oxygen transport, this protein also acts as a phenoloxidase [40], a carrier protein [41], and has a role in immune response [42]. Recent evidence shows that haemocyanin works in crayfish sclerotisation of the new exoskeleton at ecdysis [40]. Although studies have shown multiple functions for haemocyanin in crustaceans [40–42], a lack of understanding remains relating to the regulation and coordination of expression of various haemocyanin and haemoglobin genes in crustaceans, and the probability of transgenerational epigenetic inheritance in response to development and molting.

**Table 2:** Summary of proteins identified within water surrounding a molting female *M*. *rosenbergii*.

BLAST match	Species	E-value	Gene ID
Haemocyanin subunit 1	*Macrobrachium nipponense*	0	CL2258.Contig1_All
Haemocyanin	*Macrobrachium nipponense*	0	CL4434.Contig2_All
Haemocyanin	*Exopalaemon carinicauda*	0	Unigene8041_All
Chitinase 3A	*Litopenaeus vannamei*	0	CL3278.Contig1_All
Amylase	*Litopenaeus vannamei*	0	Unigene13864_All
Proteophosphoglycan ppg4	*Leishmania braziliensis*	5.00E-136	Unigene18334_All
Beta-actin	*Macrobrachium rosenbergii*	0	CL25.Contig4_All
Beta-actin	*Macrobrachium rosenbergii*	0	CL25.Contig6_All
Beta-actin	*Macrobrachium rosenbergii*	0	CL25.Contig20_All
Legumain	*Aplysia californica*	7.00E-104	Unigene43346_All
Haemocyanin	*Macrobrachium nipponense*	0	CL4434.Contig1_All
Vanin-like protein 1-like	*Eufriesea mexicana*	1.00E-24	Unigene41515_All

### Metabolite profiling of positive and negative ionization data to explore changes in the antennal gland

Classically, metabolomics experiments have used NMR- and MS-based analytical techniques to reveal the metabolite content of experimental samples. UHPLC-QToF-MS has received much attention in recent years for environmental metabolomics fingerprinting as well as in many other fields of chemical biology [12,43,44]. To extract the maximum number of metabolites within the AnG, a non-targeted extraction method was used. Methanol is widely used and is capable of extracting a large proportion of compounds other than those that are extremely polar or non-polar [44]. As an organic solvent, methanol has the additional benefit of denaturing and thus causing the precipitation of macromolecules such as proteins, as well as initiating the partition of polar analytes to the aqueous layer. Although untargeted, the choice of solvent should dictate that resultant extracts contain much of the metabolome of interest. The overall workflow for this experiment is shown in **(**S1 Fig).

### Positive mode

**Fig 3:**
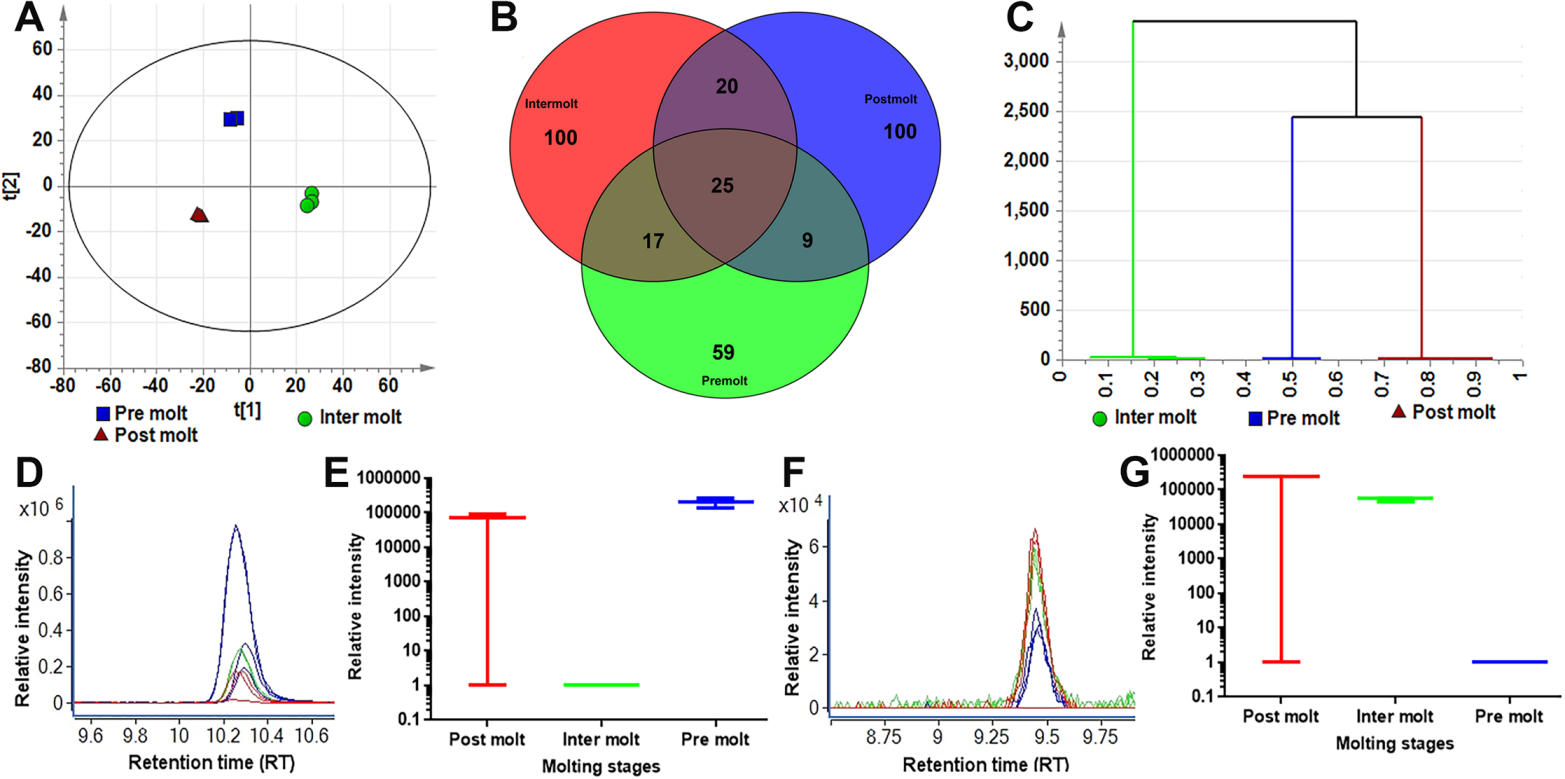
Changes of metabolic profiles in different molting stages (positive mode ionisation). (**A**) Principal component analysis (PCA) scores plot, PC1 (t[1]) versus PC2 (t[2]) showing the variation in the chemical profiles of three molting periods (green, inter molt), (blue, pre molt) and (red, post molt). Each symbol represents one *M*. *rosenbergii* antennal gland sample described by all detected features (metabolites). (**B**) Venn diagram generated from acquired positive mode LC-MS data from female AnG collected during three molting stages Premolt (green), Intermolt (red) and Postmolt (blue). (**C**) Hierarchical clustering analysis of three molting stages (plot coloured by three molting stages (**D**) Extracted ion chromatograms (EICs) of m/z 205.0971 from three molting stages, showing clear differences in the abundance of this metabolite during molting periods, (green, intermolt), (blue, premolt) and (red, postmolt). (**E**) Box-and-whisker plot of the abundance of the 205 ion in three molting stages. (**F**) Extracted ion chromatogram (EIC) of m/z 360.1909 from three molting stages, showing clear differences in the abundance of this metabolite during molting periods, (green, intermolt), (blue, premolt) and (red, postmolt) (**G**) Box-and-whisker plot of the abundance of the 360 ion in three molting stages.

LC-MS profiles were studied for changes in metabolites during the three stages. The total dataset (all extracted compounds detected within the *m/z* range of 100–1700; 577 detected features) was first evaluated using PCA to identify any outliers and assess any groupings or trends. The PCA scores plot (illustrating the relative similarities or differences of the sample extracts of the three sample groups) shows the separation of samples based on their molting stages; the scores for the first two components of the model are shown in Fig 3A. A two component PCA model explains 75.5% of the variance in the dataset. PCA reveals that the first component effectively summarises the variance relating to intermolt versus postmolt and premolt; the second component (vertical) summarises a progressive “graded” difference between postmolt versus premolt and intermolt. The variables responsible for any grouping or clusters in the data could be determined from the loadings plot (S2A Fig). To further assess compounds uniquely present to each stage, a Venn diagram plot was created from LC-MS acquired data (Fig 3B). The dendrogram obtained from this analysis shows two main clusters: (1) the intermolt subclass and (2) the other two subclasses, which divide into premolt and postmolt (Fig 3C). Each cluster, comprising one subclass, is characterised by homogeneity and is chemically distinct from the other two classes. An example of a differential metabolite is presented for the compound with 205.0971 *m/z* at RT 21 min, which discriminates the samples in the lower quadrant (Fig 3D). These findings are confirmed by extracted ion chromatogram (EIC) and box-and-whisker plot created for *m/z* 205.0971 (Fig 3D and 3E). Likewise, Fig 3F and 3G showed EIC and Box-and-whisker plot for *m/z* 360.1909 (Fig 3F and 3G). It is worthy of mention that the multivariate analysis is used to identify important/discriminatory compounds/features within the dataset and that the actual confirmation of their importance should always be achieved by extracting the representative data to ascertain the behaviour of these compounds across the sample set.

The accurate mass *m/z* values from high-resolution measurements highlighted by PCA are used to generate molecular formulae to propose putative compounds. As molecular weight increases so do the number of possible molecular formulae [45]. Compound proposals retained after both statistical and visual/manual curation was compiled as a list of accurate mass values, corresponding putative molecular formulae, RTs and IDs (S1 Table), and this list was used for future targeted analysis of the three stages.

### Negative mode

**Fig 4:**
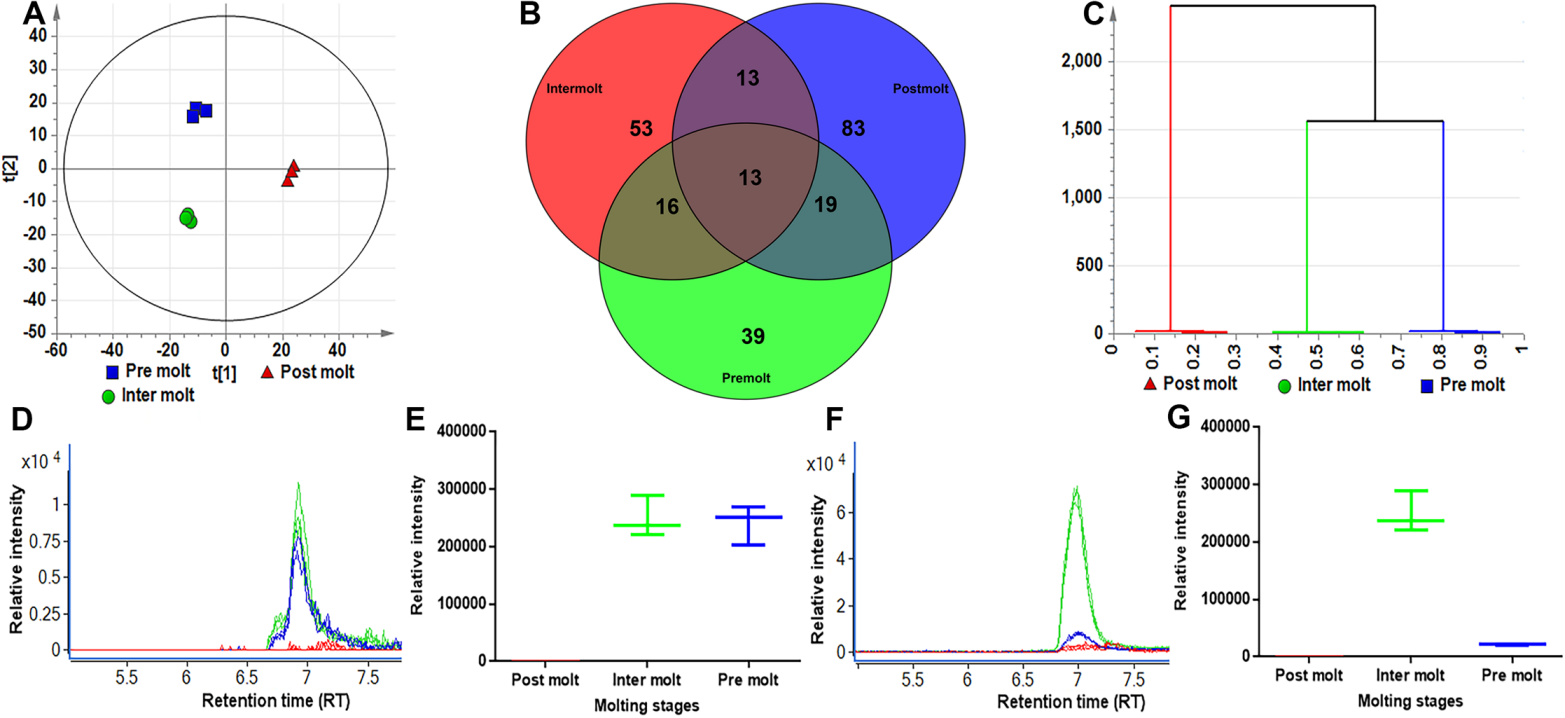
Changes of metabolic profiles in different molting stages (negative mode ionisation). (**A**) Principal component analysis (PCA) scores plot, PC1 (t[1]) versus PC2 (t[2]) showing the variation in the chemical profiles of three molting periods (green, intermolt), (blue, premolt) and (red, postmolt). Each symbol represents one AnG sample described by all detected features (metabolites). (**B**) Venn diagram generated from AnG samples from three molting stages female AnG through negative mode LC-MS data acquisition. Premolt (green), intermolt (red) and postmolt (blue). (**C**) Hierarchical clustering analysis of three molting stages (plot coloured by three molting stages (**D**) Extracted ion chromatogram (EIC) of m/z 440.2271 from three molting stages, showing clear differences in the abundance of this metabolite during molting periods, (green, intermolt), (blue, premolt) and (red, postmolt). (**E**) Box-and-whisker plot of the abundance of the 440 ion in three molting stages. (**F**) Extracted ion chromatogram (EIC) of m/z 220.1059 from three molting stages, showing clear differences in the abundance of this metabolite during molting periods, (green, intermolt), (blue, premolt) and (red, postmolt) (**G**) Box-and-whisker plot of the abundance of the 220 ion in three molting stages.

Initially, unsupervised analysis by PCA was used to identify any outliers and assess any groupings or trends in the data set. The PCA scores plot shows the separation of samples based on the molting stages; the scores for the first two component model are shown in Fig 4A. The scores plot also confirms that no technical outliers are present. A three component PCA model explains 80% of the variance in the dataset. Samples collected from premolt and intermolt show similarities in their chemical profiles, whereas samples collected during postmolt show a distinctly different pattern of metabolites. Results from the Venn diagram show the presence of unique sets of molecules in three different stages (Fig 4B). The dendrogram obtained from this analysis shows two main clusters: (1) the postmolt and the other two classes, which divide into premolt and intermolt (Fig 4C). The variables responsible for any groupings or clusters in the data can be determined from the loadings plot (S2B Fig). As an example extracted ion chromatogram (EIC) for *m/z* 440.2271 at RT 6.80 min (Fig 4D) and corresponding box-and-whisker plot (Fig 4E); EIC for *m/z* 220.1059 at RT 7.10 min (Fig 4F) and box-and-whisker plot (Fig 4G), distinguishes the samples in the loadings plot. Differences in the abundance of the compounds are also confirmed by the creation of box-and-whisker plots (Fig 4G). Analysis of the loadings plots (positive and negative mode ionisation) indicates that the variation in putatively identified compounds is the main cause of the separation between sample groups, summarized in Table 3 and S1 Table. To further delineate compounds responsible for molting stage differences, three OPLS-DA models were generated using molting stage as a classifier (S3 Fig). The resultant OPLS-DA models identified a clear separation between postmolt, premolt and intermolt. OPLS-DA provided a clearer separation and was used to define those features (variables) responsible for differences amongst the two classes related to molting. In this model, variables that were highly relevant for explaining predicted changes during molting were also identified from variable importance in the projection (VIP) and S-plot values (Table 3 and, S1 and S2 Tables).

**Table 3:** Summary of metabolites identified within female *M*. *rosenbergii* antennal gland during three stages.

Compound ID	Intermolt	Premolt	Postmolt
Arginine	✓	✓	✓
Histidine	✓	✓	✓
Leucine	−	✓	✓
Proline	✓	✓	✓
Tryptophan	—	✓	—
L-Phosphoarginine	—	✓	—
CDP-DG(16:0/20:4(5Z,8Z,11Z,14Z))	✓	—	—
DHAP(8:0)	—	—	✓
LysoPE(0:0/18:1(11Z))	✓	−	−
LysoPE(20:5(5Z,8Z,11Z,14Z,17Z)/0:0)	✓	−	−
MG(P-18:0e/0:0/0:0)	—	—	✓
PA(16:0/18:1(9Z))	✓	—	—
PC(O-16:1(11Z)/2:0)	✓	−	−
PC(16:0/0:0)	✓	−	−
PC(O-12:0/2:0)	✓	−	−
Sphingosine-1-phosphate	✓	—	—
TG(20:0/22:2(13Z,16Z)/o-18:0)	✓	−	−
Epinephrine	✓	✓	—
GABA	✓	—	✓
Serotonin	—	✓	—
Tyramine	✓	−	✓
Allo-Inositol	✓	—	—
Dibutyl disulphide	✓	—	—
Dihydro-2,4-dimethyl-6-(2-methylpropyl)-4H-1,3,5-dithiazine	—	—	✓
Isovaleric acid	✓	—	—
Juvenile hormone I	−	−	✓
Methyl farnesoate	✓	✓	−
N-Acetyl-b-glucosamine (NAG)	✓	—	—
N-acetylglucosamine-1,5-lactone (NAGL)	—	—	✓
PGE2-EA	✓	✓	—
Uric acid	−	✓	✓
2,3-Methyleneglutaric acid	✓	—	—
1,6-Dimethylnaphthalene	✓	✓	—

The amino acids arginine, proline and histidine were detected in all molt stages. Tryptophan and L-phosphoarginine were observed only in premolt, and leucine was found in premolt and postmolt. This contrasts with a previous report that stated that there was no obvious difference in amino acid content between the tiger prawn *Penaeus monodon* intermolt and premolt whole body samples [46], suggesting tissue-specific variation. In addition to metabolites, we have also identified a significant number of small peptides within the *M*. *rosenbergii* AnG (S2 Table). During postmolt, water uptake is critical to facilitate the expansion of the new, still soft exoskeleton, which eventually hardens to protect against predation. Using a Venn diagram comparative analysis, we show that 100 metabolites (positive mode acquisition) (Fig 3B) and 83 metabolites (negative mode acquisition) (Fig 4B**)** are unique to this stage. Loadings plot and S-plot analysis from pairwise OPLS-DA revealed several compounds tentatively identified during the postmolt stage. For example, we have identified two lipid molecules in the postmolt and nine in the intermolt stages (Table 3). During intermolt, a significant amount of stored energy is required to continue growth and development in crustaceans [47,48]. Studies have reported that in crustaceans, *fas* and *acly* genes enable fatty acid production during intermolt, catalysed by a lipid synthesis pathway through conversion of carbohydrates into fatty acids [49,50]. In this study, the presence of lipids in the intermolt stage indicates a requirement for these during molting, as this is the longest stage and requires more energy for metabolic processes. Several studies have reported that crustaceans utilise a significant amount of carbohydrate, lipid, fatty acids, and fat-soluble vitamins during the intermolt stage for subsequent molting and limb generation [7,49].

### Analysis of metabolite synthesis pathway genes in *M*. *rosenbergii* antennal gland

**Fig 5:**
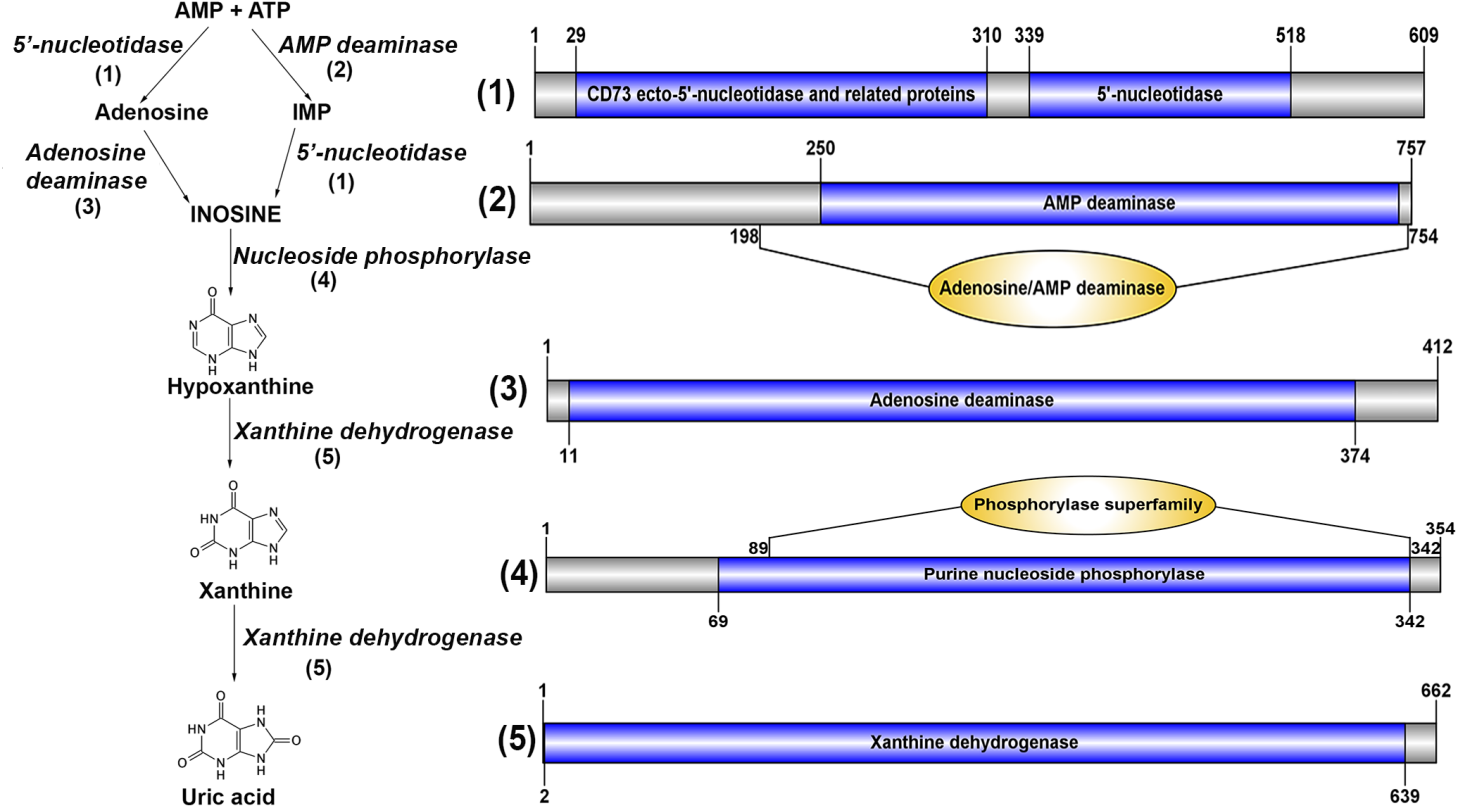
Identification of enzymes involved in uric acid biosynthesis in *M*. *rosenbergii*. Pathway for uric acid synthesis, and schematics showing enzymes (1–5) with characteristic domains found in *M*. *rosenbergii* AnG transcriptome. Biosynthetic enzymes-derived from *M*. *rosenbergii* are listed in S4 File.

Several metabolites identified within the AnG have biosynthetic pathways that have been defined in other animals, including those implicated in conspecific communication like uric acid and N-acetylglucosamine-1,5-lactone (NAGL). In crustaceans, urine contains uric acid, which can be excreted with minimal loss of water. Additionally, urates can precipitate with cations, allowing their excretion or storage, and can function as powerful antioxidants in human and molluscs [51,52]. We found uric acid is present in multiple AnG stage samples (premolt and postmolt), and our analysis of the AnG transcriptome indicated the presence of all enzyme pathway genes associated with uric acid biosynthesis (Fig 5). A role for uric acid in pheromone-induced behaviour changes has been documented, including for the marine annelid *Platynereis dumerilli* [53]. In *P*. *dumerilli*, uric acid was identified as the sperm-release pheromone that is discharged by the female congruent with the release of eggs [53]. Urine contains many nitrogenous excretory metabolites from tissues and hemolymph molecules such as ammonia, urea, uric acid, and amino acids in different crustaceans and also reported their variations in their concentrations in male and female [54]. It has also been found that some nitrogenous metabolites stimulate conspecific aesthetasc sensillae, structures reported to house pheromone-specific receptors [55]. Although we have identified uric acid in pre and postmolt AnG of *M*. *rosenbergii*, its role in signalling in this species is unknown.

**Fig 6:**
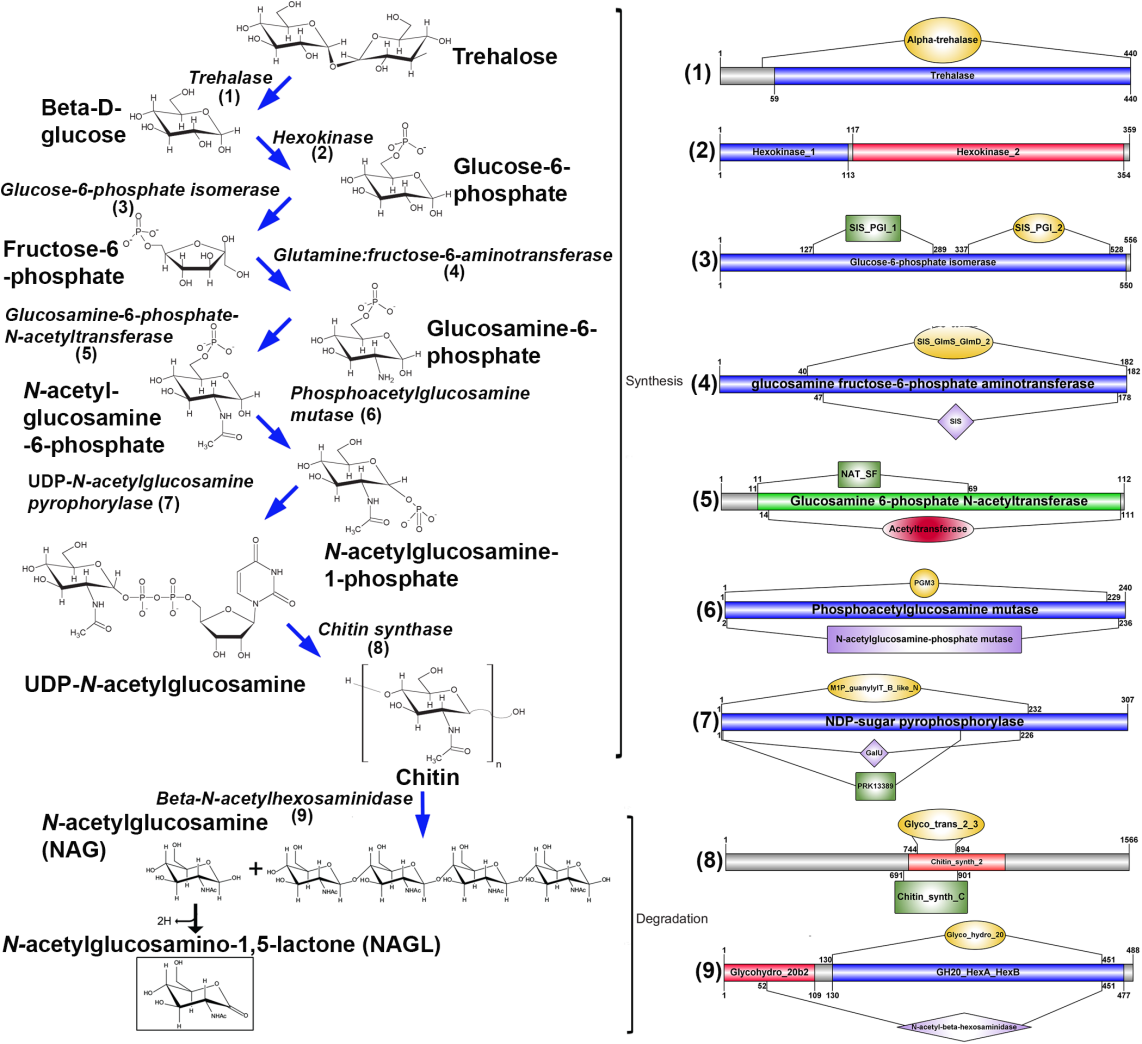
Proposed biosynthesis pathways for NAGL in *M*. *rosenbergii*. Pathway for biosynthesis and degradation of chitin and NAGL, and schematics showing enzymes with characteristic domains found in *M*. *rosenbergii* AnG transcriptome. Pathway figure modified from [12,56]. Biosynthetic enzymes-derived from *M*. *rosenbergii* are listed in S4 File.

Blue crabs in their pubertal molt stage release a sex pheromone in their urine, causing males to respond with courtship behaviour [12]. We have found the small molecule, NAGL (present in chitin biosynthesis pathway), a degradation product from the breakdown of chitin, in postmolt *M*. *rosenbergii* AnG samples. Most of the associated biosynthesis pathway enzyme genes could be identified from AnG transcriptome data (Fig 6). NAGL has previously been identified as a pheromone in urine obtained from premolt female and male blue crabs [12], demonstrating that NAGL is more abundant in premolt female and male urine than in postmolt or juveniles. NAGL (logP = -3.04 (ACD/Labs Percepta Platform–PhysChem module for compound *N*-((3*R*,4*R*,5*S*,6*R*)-4,5-dihydroxy-6-(hydroxymethyl)-2-oxotetrahydro-2*H*-pyran-3-yl)acetamide) is more polar than NAG (logP = -2.48 (*N*-((2*S*,3*R*,4*R*,5*S*,6*R*)-2,4,5-trihydroxy-6-(hydroxymethyl)tetrahydro-2*H*-pyran-3-yl)acetamide) and would thus be more water soluble, ensuring a more pronounced transmission. The *cbm* gene in highly expressed in the molting crab, which is primarily thought to have an active role in chitin biosynthesis, allowing for rapid chitin synthesis to recover their outer shell [7]. Cannibalistic crustaceans may sense NAGL, rendering the source individuals open to attack. In our study the metabolite collection was not only from the AnG but also from the surrounding water, enabling the sampling of small molecules secreted into the environment as well as from the AnGs themselves. It is possible that female *M*. *rosenbergii* store NAGL within the AnG postmolt rather than secrete it.

Other metabolites were identified whose pathways and functions have been well studied in crustaceans, with particular reference to growth and reproduction [57–59]. The synthesis of the juvenile hormone (JH) equivalent in crustaceans, methyl farnesoate (MF), starts in the mandibular organs that secrete its precursor, farnesoic acid (FA). FA is then converted to MF in the hemolymph [60]. In insects, JH regulates metamorphosis and gametogenesis [60,61]. In our metabolomics study, the presence of MF and JH I in *M*. *rosenbergii* AnG have been tentatively identified. Moreover, we have identified the major enzymes for MF and JH I biosynthesis (S4 Fig). In *M*. *rosenbergii*, haemolymph MF fluctuates during the molt cycle [9,34]. The highest levels occur during early premolt, apparently preceding the ecdysteroid peak, while the lowest levels have been reported during late premolt stages [34]. However, no role for JH I or MF has been explored in crustaceans.

### Molecular characterization and protein-ligand modelling of *M*. *rosenbergii* hemocyanin with NAG and NAGL

**Fig 7:**
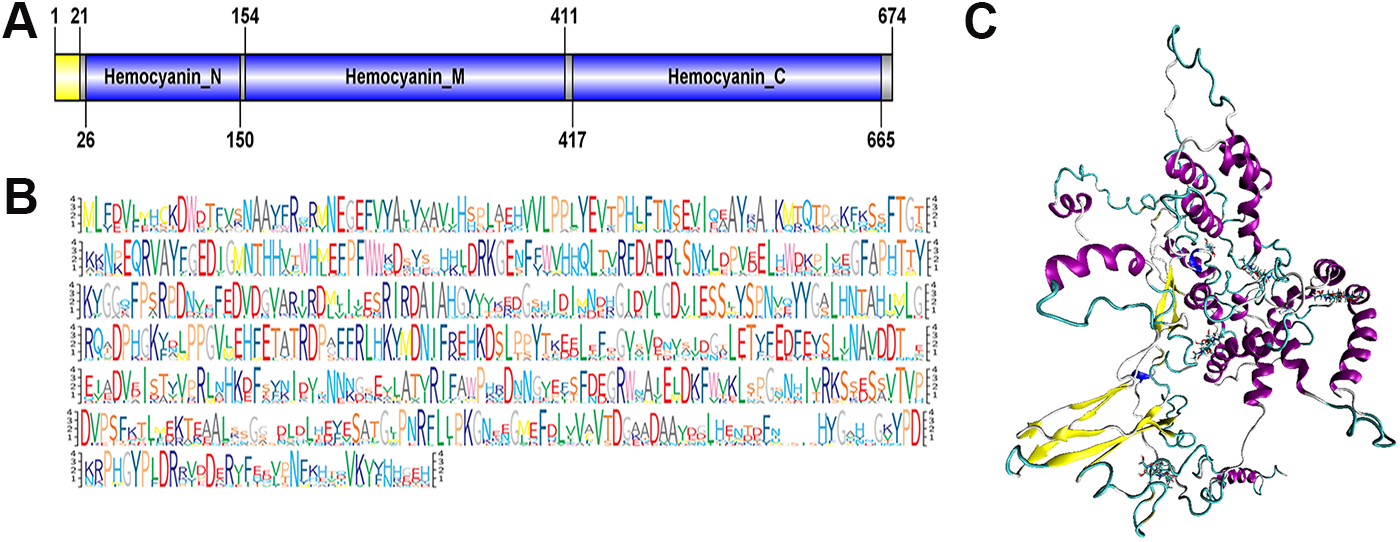
Molecular characterization of *M*. *rosenbergii* haemocyanin subunit 1. (**A**) Schematic diagram showing the general the general organisation of *M*. *rosenbergii* haemocyanin. Signal peptide (yellow) and conserved domains (blue). (**B**) Sequence logo representation of multiple sequence alignment for *M*. *rosenbergii* with other species. Gene sequences are provided in (S5 File). **(C)** Predicted structure of *M*. *rosenbergii* hemocyanin subunit 1. Secondary structures: blue, 3–10 helix; purple, alpha-helix; cyan, turn and white, random coil.

We had found haemocyanin subunit 1 to be relatively abundant within the AnG at premolt (see Fig 2) and it is also present in the water surrounding molted female prawns (Table 2). Domain analysis shows that *M*. *rosenbergii* haemocyanin subunit 1 contains all conserved domains typical for haemocyanin (Fig 7A), and the overall amino acid composition is highly conserved with other species, based on comparative multiple sequence alignment (Fig 7B), where most conservation exists with the *Macrobrachium nipponese*, then shrimp species that belong to the family Atyidae. To gain further insight into the prawn haemocyanin subunit 1, its 3D protein structure was predicted using the SWISS-MODEL [62], including alpha helical regions that are likely critical for ligand binding (Fig 7C and S5 Fig).

**Fig 8:**
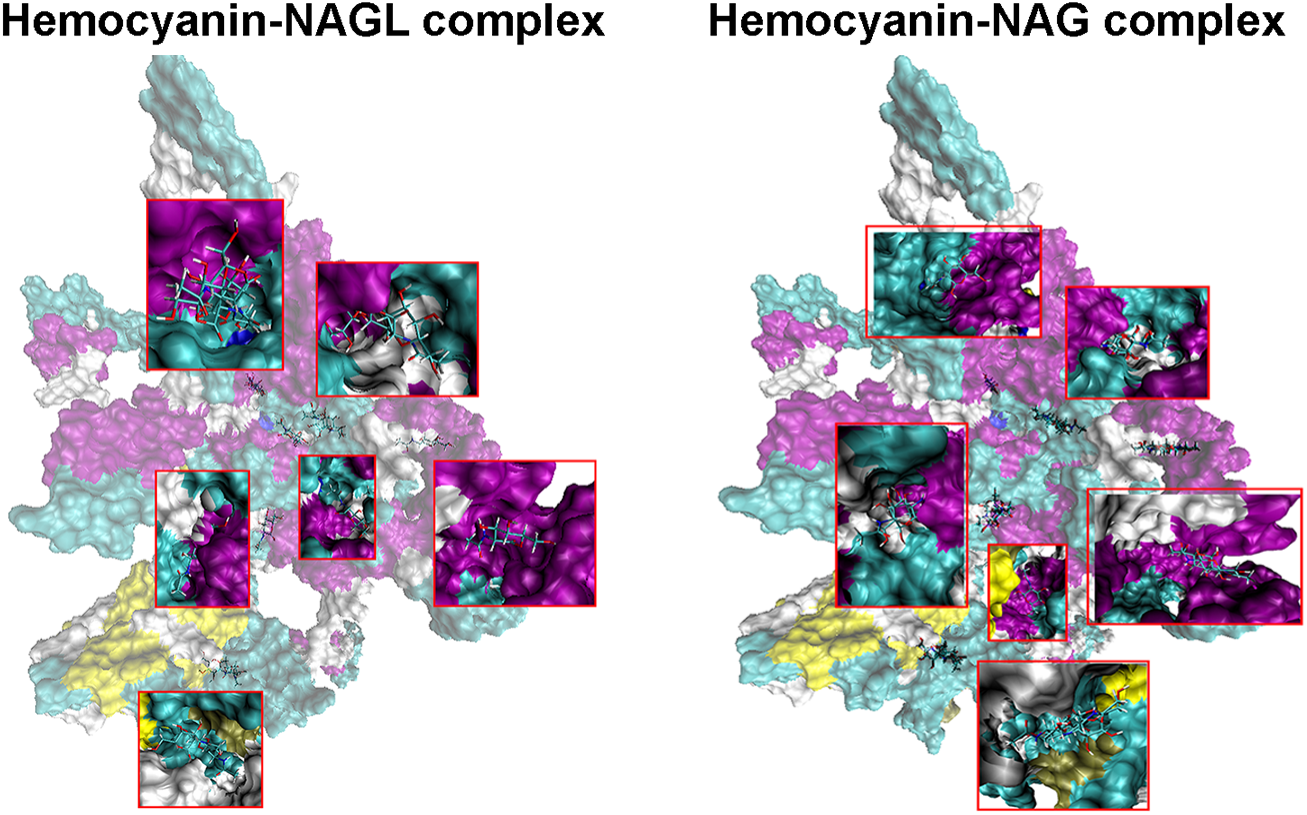
Proposed binding between hemocyanin subunit 1 and NAG/NAGL. A space filling structure of haemocyanin subunit 1-NAG complex, with binding sites enlarged (left). A space filling structure of haemocyanin subunit 1-NAGL complex, with binding sites enlarged (right). Secondary structures: yellow, 3–10 helix; purple, alpha-helix; cyan, turn and white, random coil.

Our metabolomics analysis had similarly identified NAG within intermolt stage AnG (see Table 3), so we next explored the possibility that the *M*. *rosenbergii* haemocyanin subunit 1 could bind NAG, as well as NAGL. *In silico* protein-ligand binding analysis illustrates that the prawn haemocyanin subunit 1 could act as a carrier for either of NAG or NAGL (Fig 8); binding sites were observed for both metabolites based on theoretical constructs, as listed in (S6 File), with the relevant residues. As previously mentioned, in crabs NAGL has been found in the urine at intermolt and premolt stages [12]. The absence of NAGL in the *M*. *rosenbergii* intermolt stage AnG may be because 1) our metabolite analysis was unable to detect NAGL, 2) NAGL was released from other sites at intermolt, not the AnG, or 3) NAGL is not used as a pheromone in this prawn (and possibly other prawn species). We did, however, find NAGL within the AnG at postmolt stage.

## Conclusions

In summary, the worldwide distribution of *M*. *rosenbergii*, together with its commercial significance and well-defined social structure, ensure that this species could be a leading model for crustacean pheromone studies. In this study, multi-omics analyses were carried out to determine potential biomolecules present and released by the female *M*. *rosenbergii* AnG. Our *in silico* analysis of the AnG transcriptome predicted those proteins secreted, while proteomic analysis has confirmed some that are secreted differentially between stages of intermolt, premolt and posmolt, and that the hemocyanin subunit 1 is also present in the surrounding water of molting females. Using untargeted metabolomics of the AnG, we have identified groups of molecules based on their accurate masses i.e. amino acids, lipids, neurotransmitters, JH I, NAG, lactone and some small peptides. We show that some metabolites implicated in conspecific communication may be synthesised in the AnG, supported by the presence of biosynthesis enzymes. Predicted protein-ligand models indicate that NAG and NAGL bind to haemocyanin subunit 1, which may be a requirement for endogenous and exogenous transport. Future studies will focus on the analysis of NAG and NAGL with, and without haemocyanin subunit 1, to assess its potential importance as a pheromone in *M*. *rosenbergii*.

## Supporting information

S1 Fig Schematic representation of method for identifying metabolites (small molecules) during three stages in *Macrobrachium rosenbergii*. (TIF)

S2 Fig Loadings plot originated from principal component analysis. Inspection of the 2-D loadings plot for PC1 vs. PC2 reveals the variables responsible for the spatial arrangement of samples in (A) positive mode ionisation (B) Negative mode ionisation.
(TIF)

S3 Fig Pair-wise comparison between three molting stages. (A) Orthogonal projection to latent structures-discriminant analysis (OPLS-DA) scores plot of predictive components t[1] versus t[2] showing the supervised separation between the two sample classes based upon molting time period (green, intermolt and red, postmolt). The ellipse shown in A represent the Hotelling’s T2 95% confidence interval for the multivariate data. Data are log10 transformed and mean centred. (B) OPLS-DA analysis of intermolt and premolt stages (green, intermolt and blue, premolt). (C) OPLS-DA analysis of postmolt and premolt.
(TIF)

S4 Fig Proposed biosynthesis pathways juvenile hormone I in *M*. *rosenbergii*. Pathway for juvenile hormone I synthesis and schematics showing enzymes with characteristic domains found in *M*. *rosenbergii* antennal gland transcriptome. Biosynthetic enzymes-derived from *M*. *rosenbergii* were listed in **S5 File.**
(TIF)

S5 Fig Protein-ligand binding assay. (A) 3D structure of arthropod haemocyanin (B) *M*. *rosenbergii* haemocyanin. (C) Potential energy as a function of MD simulation time (top). Backbone rmsd during the same MD, compared to the lowest-energy conformation (bottom).
(TIF)

S1 File Unigene sequences and annotation information of all *M*. *rosenbergii* sequences. (XLS)

S2 File *In silico* analysis of the AnG transcriptome to predict secreted proteins. (XLS)

S3 File Differentially expressed proteins between three molting stages. (XLS)

S4 File Uric acid, NAGL and juvenile hormone I biosynthetic enzymes-derived from *M*. *rosenbergii*. (TXT)

S5 File Gene sequences used for phylogenetic analysis. (TXT)

S6 File Binding sites were observed for NAG and NAGL based on theoretical constructs with the relevant residues. (DOCX)

S1 Table Details of metabolites identified from *M*. *rosenbergii* antennal gland during three molting stages. (DOCX)

S2 Table Lists of small peptides identified from *M*. *rosenbergii* antennal gland. (DOCX)
