# Supplementary figures and images for: Biomolecular changes that occur in the antennal gland of the giant freshwater prawn (Machrobrachium rosenbergii)
Source: PLoS One. 2017 Jun 29;12(6):e0177064. doi: 10.1371/journal.pone.0177064 (PMC5490968; doi:10.1371/journal.pone.0177064)

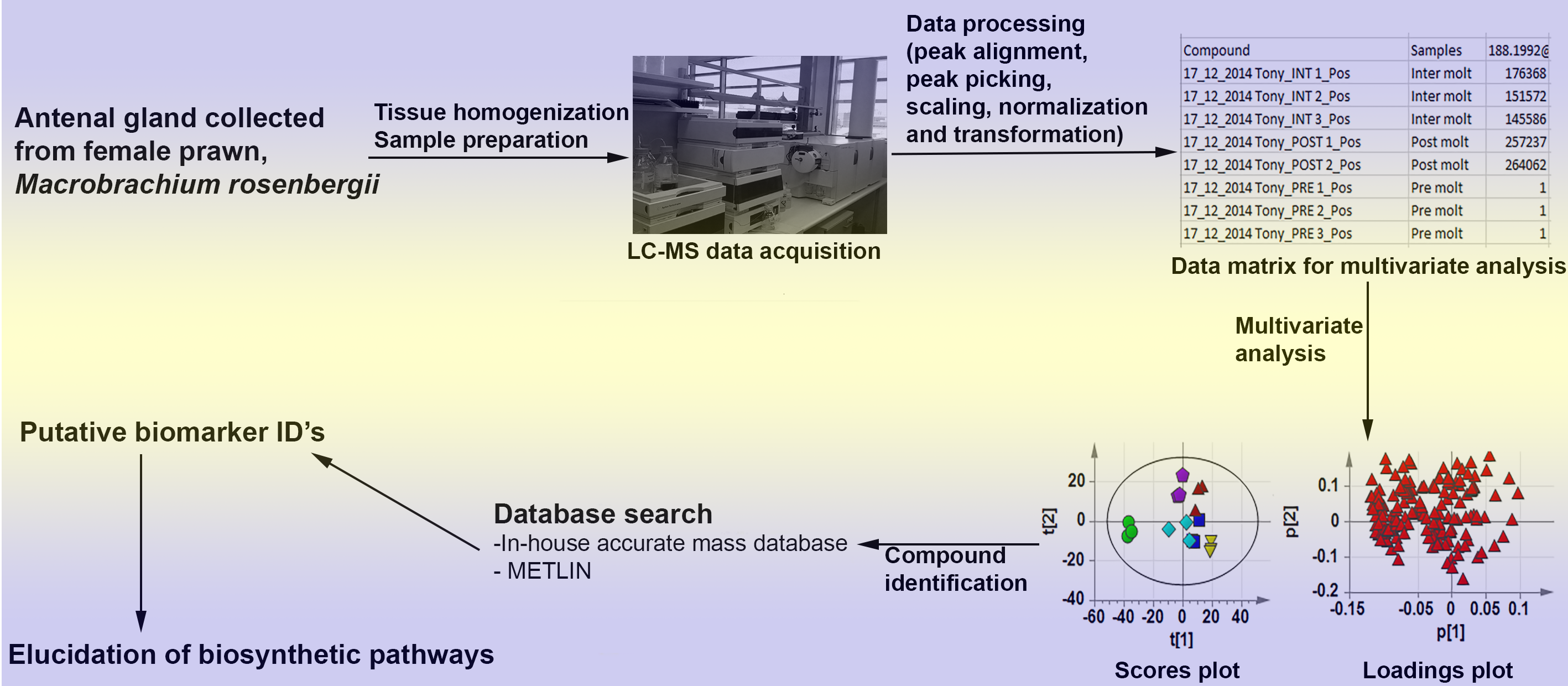

Supplement: S1 Fig — (TIF) [file pone.0177064.s001.tif]

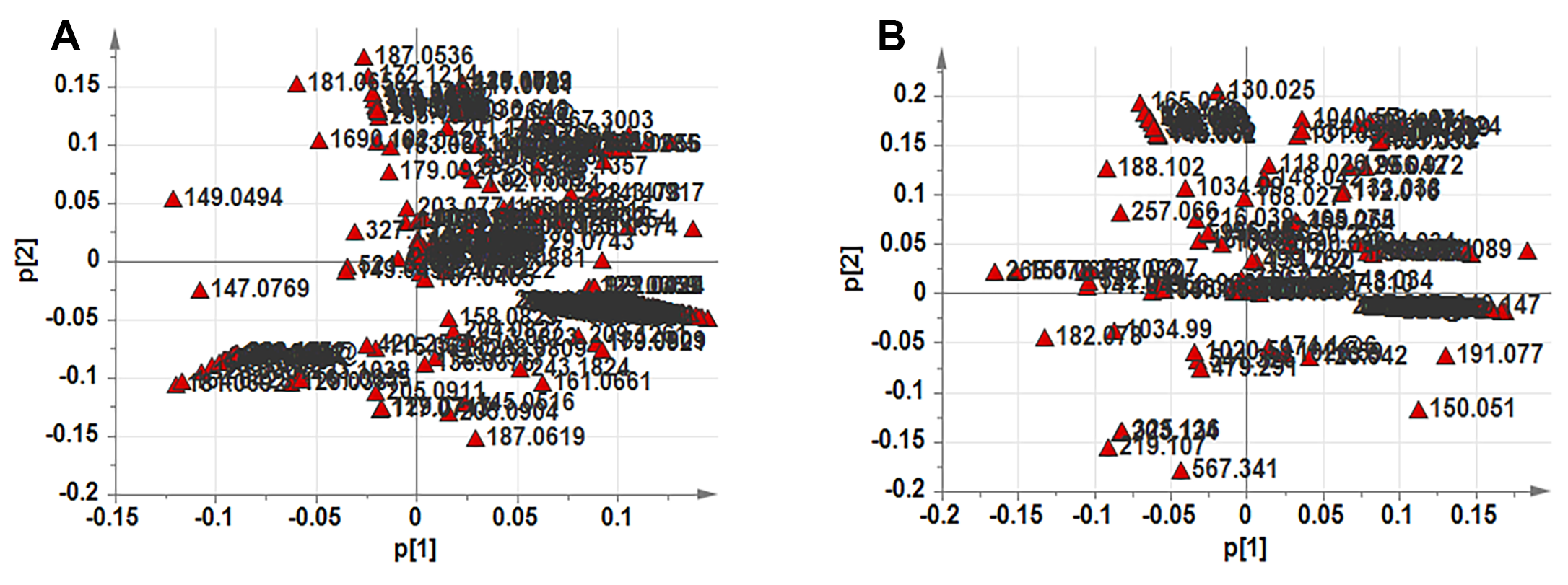

Supplement: S2 Fig — Inspection of the 2-D loadings plot for PC1 vs. PC2 reveals the variables responsible for the spatial arrangement of samples in (A) positive mode ionisation (B) Negative mode ionisation. (TIF) [file pone.0177064.s002.tif]

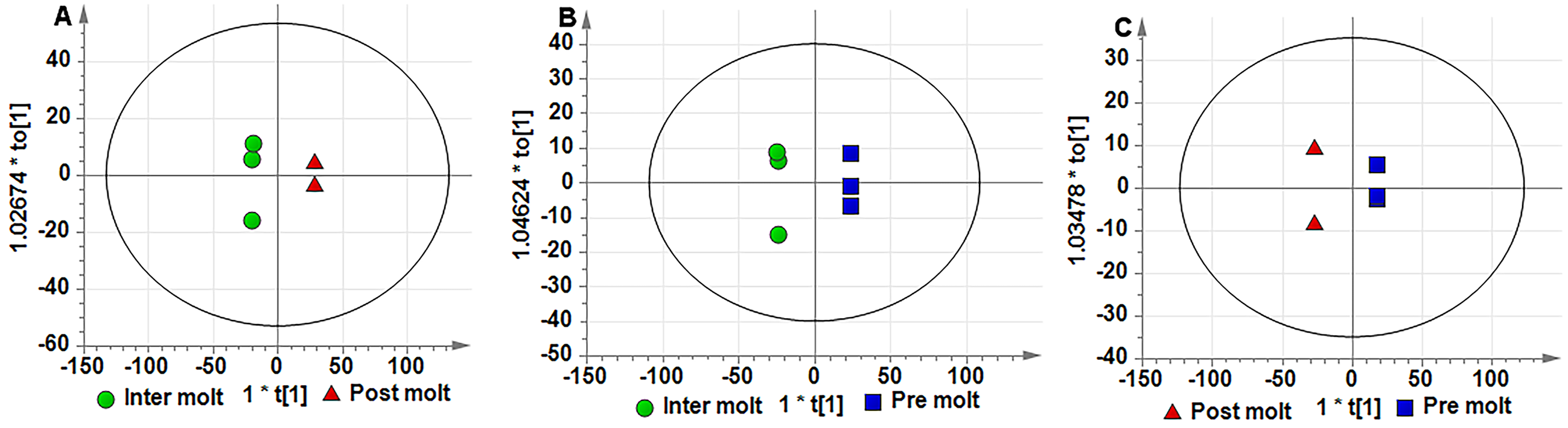

Supplement: S3 Fig — (A) Orthogonal projection to latent structures-discriminant analysis (OPLS-DA) scores plot of predictive components t[1] versus t[2] showing the supervised separation between the two sample classes based upon molting time period (green, intermolt and red, postmolt). The ellipse shown in A represent the Hotelling’s T2 95% confidence interval for the multivariate data. Data are log10 transformed and mean centred. (B) OPLS-DA analysis of intermolt and premolt stages (green, intermolt and blue, premolt). (C) OPLS-DA analysis of postmolt and premolt. (TIF) [file pone.0177064.s003.tif]

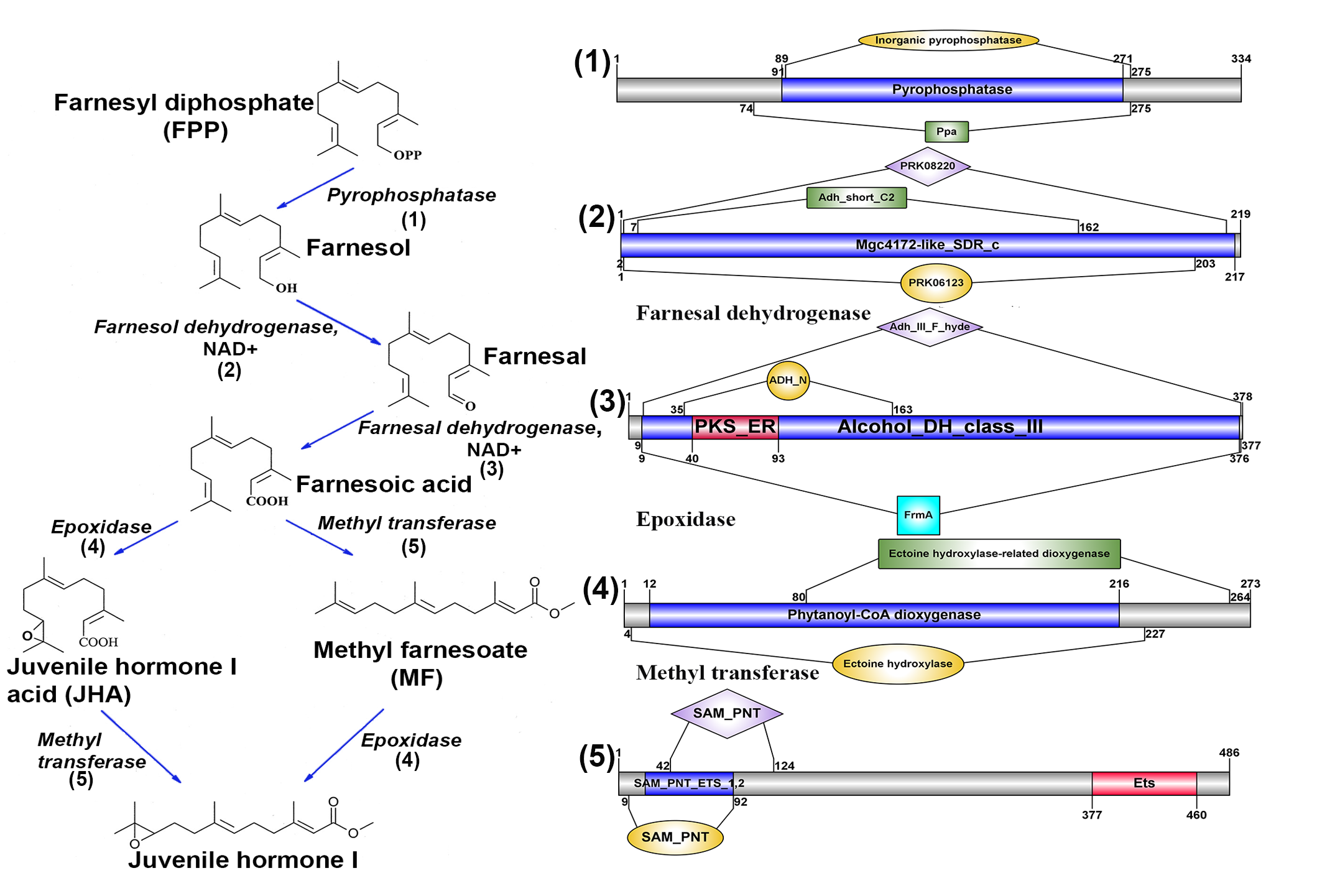

Supplement: S4 Fig — Pathway for juvenile hormone I synthesis and schematics showing enzymes with characteristic domains found in M. rosenbergii antennal gland transcriptome. Biosynthetic enzymes-derived from M. rosenbergii were listed in S5 File. (TIF) [file pone.0177064.s004.tif]

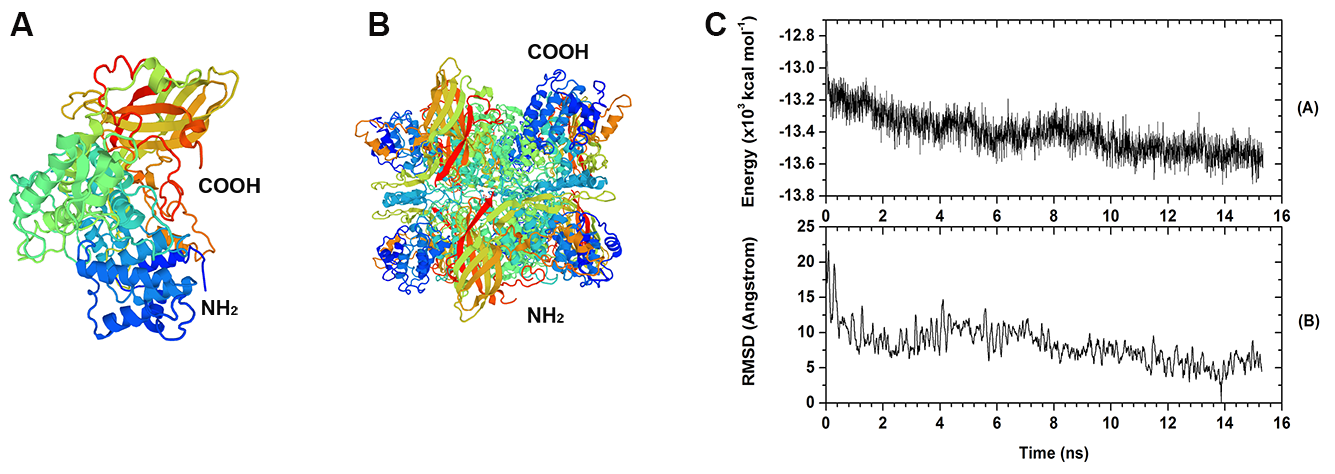

Supplement: S5 Fig — (A) 3D structure of arthropod haemocyanin (B) M. rosenbergii haemocyanin. (C) Potential energy as a function of MD simulation time (top). Backbone rmsd during the same MD, compared to the lowest-energy conformation (bottom). (TIF) [file pone.0177064.s005.tif]
