# Supplementary material for: Biomolecular changes that occur in the antennal gland of the giant freshwater prawn (Machrobrachium rosenbergii)
Source: PLoS One. 2017 Jun 29;12(6):e0177064. doi: 10.1371/journal.pone.0177064 (PMC5490968; doi:10.1371/journal.pone.0177064)
Supplement: S6 File — (DOCX) [file pone.0177064.s011.docx]

| **Binding site** | **Sequence segment relevant to binding site** |
| --- | --- |
| 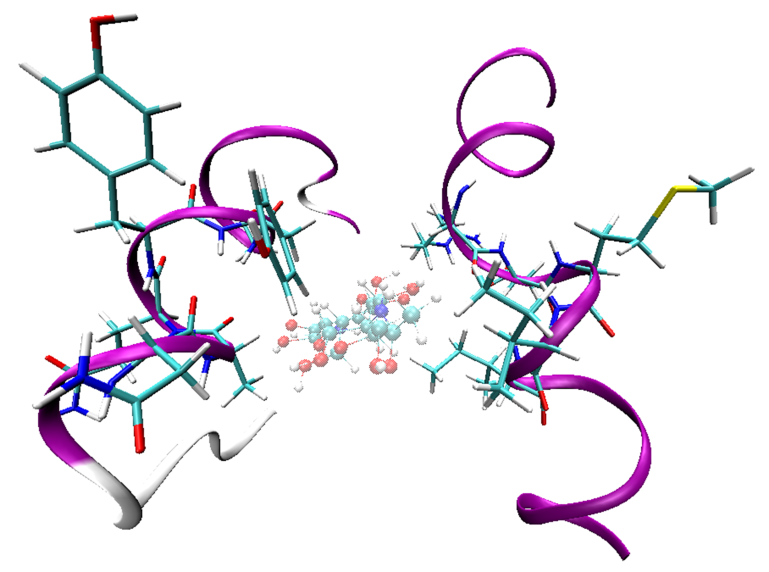 | Asn354 - Gly359 and Arg305 - ILE309 |
| 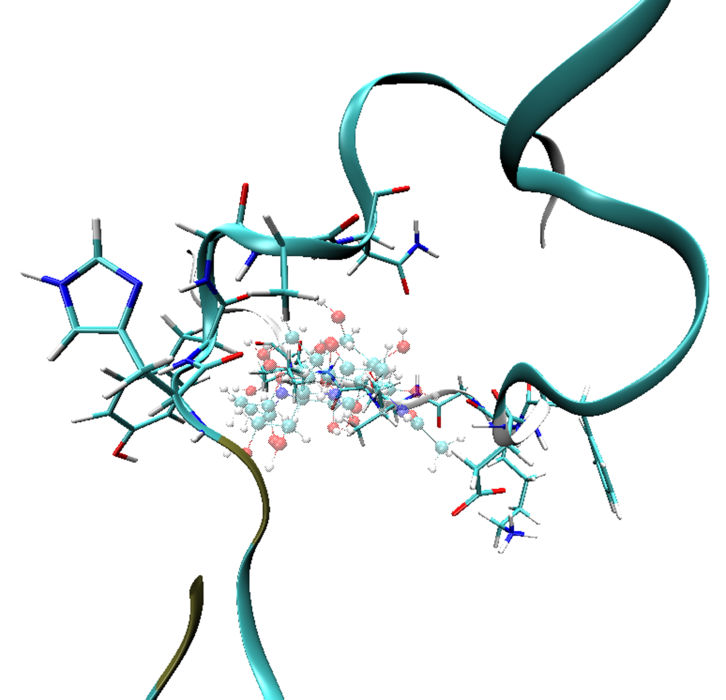 | Asp425 – Val431 and His624 – Asn628 |
| 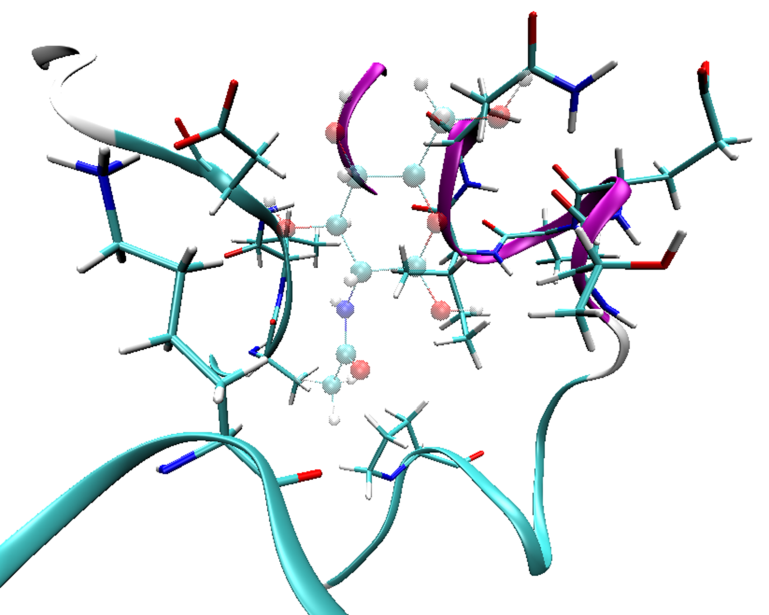 | Lys87, Pro160, Thr166 – Gln170 |
| 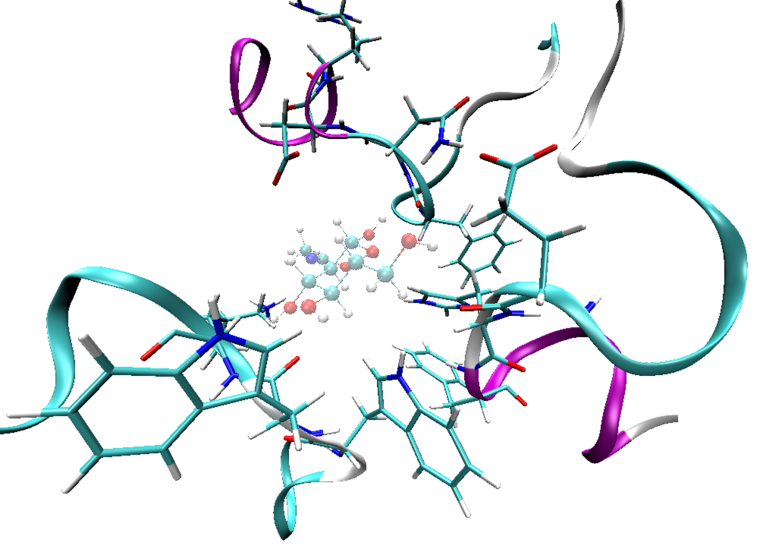 | Phe93 – Arg96, Trp222 - Lys224 and Glu387 - Phe389 |
| 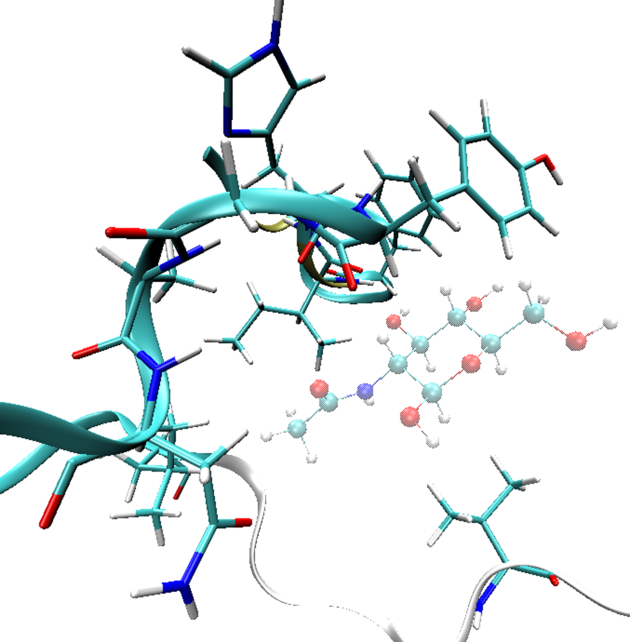 | Val429, Leu424 and Phe622 – Asn628 |
| 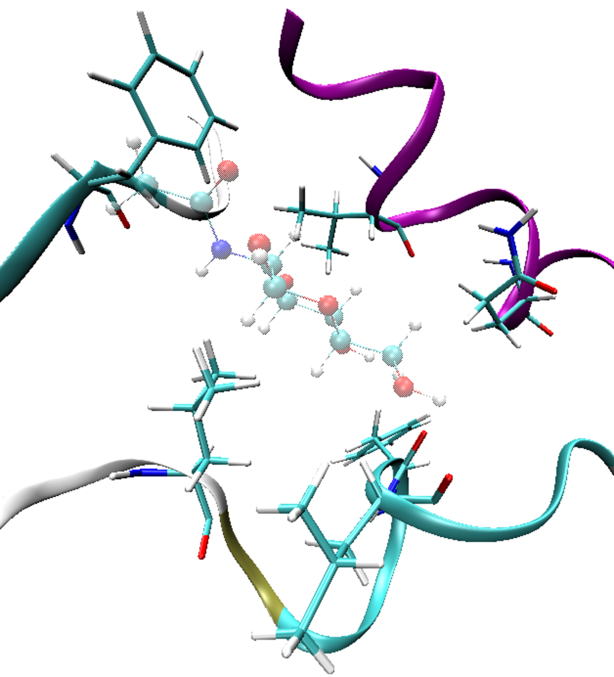 | Phe219, Val241, Gln244, Leu 582, Phe586 and Leu587 |
| 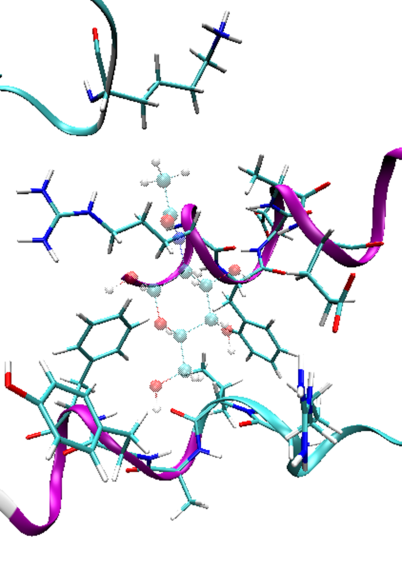 | Arg198 – Phe202, Arg248 – Glu252 and Lys590 |
