# Supplementary material for: Biomolecular changes that occur in the antennal gland of the giant freshwater prawn (Machrobrachium rosenbergii)
Source: PLoS One. 2017 Jun 29;12(6):e0177064. doi: 10.1371/journal.pone.0177064 (PMC5490968; doi:10.1371/journal.pone.0177064)
Supplement: S1 Table — (DOCX) [file pone.0177064.s012.docx]

**Supporting Table S1:** Details of metabolites identified from *M. rosenbergii* antennal gland during three molting stages.

| **Molecular Formula** | **Compound ID** | **Adduct** | **Mass** | **RT (min)** | **Diff (ppm)** |
| --- | --- | --- | --- | --- | --- |
|  |  |  |  |  |  |
| **C_6_H_12_O_6_** | Allo-Inositol | [M+H] | 181.0703 | 9.71 | 0.031 |
| **C_8_H_18_S_2_** | Dibutyl disulfide | [M+H] | 179.0921 | 7.92 | 0.001 |
| **C_22_H_37_NO_5_** | PGE2-EA | [M+H] | 395.2671 | 13.99 | 0.0373 |
| **C_12_H_12_** | 1,6-Dimetylnapthalene | [M+H] | 156.0939 | 10.06 | 0.0003 |
| **C_21_H_42_O_3_** | MG(P-18:0e/0:0/0:0) | [M+2H] | 172.1214 | 9.40 | 0.0425 |
| **C_11_H_21_O_7_P** | DHAP(8:0) | [M+2H] | 464.2635 | 9.45 | 0.001 |
| **C_26_H_52_NO_7_P** | PC(O-16:1(11Z)/2:0) | [M+H] | 522.3538 | 19.54 | 0.003 |
| **C_8_H_13_NO_6_** | N-acetylglucosamine-1,5-lactone | [M+H] | 220.0826 | 7.61 | 0.004 |
| **C_37_H_71_O_8_P** | PA(16:0/18:1(9Z)) | [M+2H] | 674.4886 | 12.84 | 0.0222 |
| **C_48_H_81_N_3_O_15_P_2_** | CDP-DG(16:0/20:4(5Z,8Z,11Z,14Z)) | [M+H] | 1001.5142 | 13.21 | 0.0354 |
| **C_50_H_73_N_15_O_11_** | Bradykinin | [M+H] | 1059.5925 | 13.76 | 0.0313 |
| **C_19_H_42_NO_5_P** | Sphingosine 1-phosphate | [M+H] | 790.4900 | 13.99 | 0.0168 |
| **C_9_H_13_NO_3_** | Epinephrine | [M+H] | 183.0901 | 9.33 | 0.041 |
| **C_4_H_9_NO_2_** | GABA | [M-H] | 102.0559 | 6.87 | 0.003 |
| **C_22_H_46_NO_7_P** | PC(O-12:0/2:0) | [M+H] | 468.3075 | 15.25 | 0.002 |
| **C_11_H_12_N_2_O_2_** | Tryptophan | [M+H] | 205.0971 | 6.76 | 0.006 |
| **C_10_H_12_N_2_O** | Serotonin | [M+H] | 176.0921 | 10.17 | 0.016 |
| **C_11_H_12_N_2_O_2_** | L-tryptophan | [M-H] | 203.0808 | 10.11 | 0.005 |
| **C_63_H_120_O_5_** | TG(20:0/22:2(13Z,16Z)/o-18:0) | [M-H] | 955.9554 | 7.46 | 0.049 |
| **C_5_H_4_N_3_O_3_** | Uric acid | [M-H] | 167.0212 | 7.19 | 0.006 |
| **C_6_H_15_N_4_O**_5_P | L-Phosphoarginine | [M+H] | 255.0852 | 9.99 | 0.0199 |
| **C_9_H_19_NS_2_** | Dihydro-2,4-dimethyl-6-(2-methylpropyl)-4H-1,3,5-dithiazine | [M-H] | 205.0950 | 10.17 | 0.004 |
| **C_6_H_16_O_2_** | Methyl farnesoate | [M+H] | 251.1937 | 7.31 | 0.002 |
| **C_18_H_30_O_3_** | Juvenile hormone I | [M+H] | 294.2194 | 7.31 | 0.002 |
| **C_7_H_8_O_4_** | 2,3-Methyleneglutaric acid | [M-H] | 156.0422 | 6.37 | 0.0209 |
| **C_15_H_10_O_2_** | Isovaleric acid | [M-2H] | 203.1244 | 6.73 | 0.0044 |
| **C_8_H_16_N_2_O_5_** | N-Acetyl-b-glucosaminylamine | [M-H] | 220.1059 | 8.48 | 0.0046 |
| **C_6_H_14_N_4_O_2_** | Arginine | [M+H] | 175.1191 | 6.54 | 0.002 |
| **C_8_H_11_NO** | Tyramine | [M+H] | 120.0808 | 9.46 | 0.009 |
| **C_5_H_9_NO_2_** | Proline | [M+H] | 116.0703 | 6.58 | 0.004 |
| **C_25_H_42_NO_7_P** | LysoPE(20:5(5Z,8Z,11Z,14Z,17Z)/0:0) | [M-H] | 498.2555 | 22.36 | 0.007 |
| **C_6_H_13_NO_2_** | Leucine | [M+H] | 132.1019 | 9.42 | 0.0098 |
| **C_6_H_9_N_3_O_2_** | Histidine | [M+H] | 156.0754 | 6.55 | 0.009 |
| **C_24_H_50_NO_7_P** | PC(16:0/0:0) | [M+H] | 496.3386 | 18.28 | 0.002 |

Formula: the molecular formula of the compound; Compound ID: Compound tentatively identified through accurate mass LC-QToF-MS analysis and database search; Adduct: an adduct is a product of a direct addition of two or more distinct molecules, for example M+H or M-H; Mass: the neutral mass of a compound; RT(min): the retention time of a compound; Diff (ppm): the difference between the observed mass and the value from the database (in parts per million).
