# Supplementary material for: Biomolecular changes that occur in the antennal gland of the giant freshwater prawn (Machrobrachium rosenbergii)
Source: PLoS One. 2017 Jun 29;12(6):e0177064. doi: 10.1371/journal.pone.0177064 (PMC5490968; doi:10.1371/journal.pone.0177064)
Supplement: S2 Table — (DOCX) [file pone.0177064.s013.docx]

**Table S2.** Lists of small peptides identified from *M. rosenbergii* antennal gland

| **Molecular Formula** | **Compound ID** | **Adduct** | **Mass** | **RT (min)** | **Diff (ppm)** |
| --- | --- | --- | --- | --- | --- |
|  |  |  |  |  |  |
| **C_18_H_24_N_4_O_7_** | Ala Asp Phe Gly | [M+2H] | 408.1645 | 7.07 | 0.0004 |
| **C_20_H_36_N_8_O_8_S** | Asp Met Gln Arg | [M+2H] | 548.2377 | 8.94 | 0.003 |
| **C_22_H_34_N_8_O_7_S** | Cys Asn Arg Tyr | [M+2H] | 554.2271 | 9.44 | 0.032 |
| **C_26_H_30_N_4_O_10_** | Asp Asp Phe Tyr | [M+2H] | 558.1948 | 10.26 | 0.004 |
| **C_23_H_46_N_10_O_6_** | Lys Lys Gln Arg | [M+2H] | 558.3602 | 11.54 | 0.02 |
| **C_17_H_32_N_6_O_6_** | Ala Ala Lys Gln | [M+2H] | 416.2383 | 11.59 | 0.006 |
| **C_20_H_33_N_7_O_5_** | Ala His Lys Pro | [M+2H] | 452.2602 | 11.91 | 0.013 |
| **C_10_H_20_N_2_O_4_** | Leu Thr | [M+H] | 232.1423 | 7.10 | 0.002 |
| **C_16_H_30_N_4_O_4_** | Lys pro Val | [M+2H] | 342.2267 | 9.40 | 0.004 |
| **C_23_H_36_N_4_O_6_** | Ala Ile Val Tyr | [M+2H] | 464.2635 | 9.45 | 0.001 |
| **C_15_H_28_N_4_O_7_S** | Ala Met Ser Thr | [M+2H] | 408.1679 | 6.76 | 0.0001 |
| **C_23_H_36_N_4_O_6_** | Ala Ile Val Tyr | [M+2H] | 464.2635 | 9.45 | 0.0425 |
| **C_7_H_14_N_2_O_4_S** | Cys Thr | [M+H] | 222.0674 | 10.05 | 0.0323 |
| **C_17_H_24_N_6_O_3_** | Arginyl-Tryptophan | [M+Na] | 360.1909 | 6.87 | 0.0323 |
| **C_9_H_15_N_3_O_5_** | Asparaginyl-Hydroxyproline | [M+Na] | 245.1011 | 8.68 | 0.0094 |
| **C_6_H_12_N_2_O_5_** | Serinyl-Serine | [M+H] | 192.0746 | 6.82 | 0.0103 |
| **C_11_H_22_N_2_O_3_S** | Methionyl-Leucine | [M+H] | 262.1351 | 9.41 | 0.0061 |
| **C_11_H_20_N_3_O_5_** | Glutamyl-Lysine | [M+H] | 297.1295 | 9.45 | 0.0070 |
| **C_9_H_19_N_3_O_3_S** | Cysteinyl-Lysine | [M+H] | 250.1219 | 10.71 | 0.0029 |
| **C_8_H_15_N_3_O_5_** | Threoninyl-Asparagine | [M+Cl] | 233.2218 | 7.21 | 0.0012 |
| **C_20_H_32_N_4_O_7_** | Pro Asp Leu Pro | [M-2H] | 294.2195 | 7.31 | 0.002 |
| **C_17_H_23_N_3_O_4_** | Pro Ala Phe | [M+Cl] | 333.1689 | 6.97 | 0.0032 |
| **C_13_H_24_N_4_O_8_** | Thr Ala Ser Ser | [M-2H] | 364.1594 | 7.56 | 0.0043 |
| **C_16_H_26_N_6_O_7_S** | Cys His Ser Thr | [M-2H] | 446.1584 | 7.37 | 0.001 |
| **C_22_H_34_N_6_O_7_** | Ala Lys Asn Tyr | [M-2H] | 494.2489 | 9.27 | 0.004 |
| **C_17_H_26_N_6_O_6_** | Ala His Pro Ser | [M-2H] | 410.1914 | 10.20 | 0.0032 |
| **C_13_H_24_N_4_O_8_** | Thr Ser Ser Ala | [M-2H] | 364.1594 | 6.42 | 0.025 |
| **C_20_H_24_N_4_O_6_** | Trp Pro Asp | [M-H] | 416.1696 | 6.80 | 0.012 |
| **C_16_H_26_N_6_O_7_S** | Asp Ile Pro Pro | [M-2H] | 446.1584 | 7.37 | 0.003 |
| **C_13_H_24_N_4_O_5_S** | Asp Ile Pro Pro | [M-2H] | 348.1467 | 7.56 | 0.002 |
| **C_10_H_15_N_5_O_4_** | Asparaginyl-Histidine | [M-H] | 268.1051 | 7.21 | 0.0264 |
| **C_10_H_15_N_2_O_6_** | Glutamyl-Hydroxyproline | [M-H] | 440.2271 | 8.48 | 0.0274 |
| **C_16_H_26_N_6_O_7_S** | Cys His Ser Thr | [M-2H] | 446.1584 | 7.37 | 0.004 |
| **C_22_H_34_N_6_O_7_** | Ala Lys Asn Tyr | [M-2H] | 494.2489 | 9.27 | 0.001 |
